# Associations between lifestyle, genetic risk for Alzheimer's disease, and longitudinal brain atrophy in the UK Biobank (*N* = 3265)

**DOI:** 10.1002/dad2.70470

**Published:** 2026-08-31

**Authors:** Yating You, Laura M. Lyall, Frederick K. Ho, Claire E. Hastie, Donald M. Lyall

**Affiliations:** ^1^ School of Health and Wellbeing University of Glasgow Glasgow Scotland UK

**Keywords:** *APOE* ε4, brain structure, cohort study, lifestyle, longitudinal atrophy

## Abstract

**INTRODUCTION:**

The associations of modifiable daily lifestyle variables (e.g., smoking, diet, alcohol intake, sedentary behavior, and physical activity) with structural brain atrophy, and interaction with apolipoprotein E (*APOE)* ε4 genetic risk, remain unclear.

**METHODS:**

Among 3265 UK Biobank participants with repeated magnetic resonance imaging (MRI) data (mean follow‐up 2.6 years), we assessed the relations between lifestyle, *APOE* ε4 genotype, and volumetric changes in 15 structural brain phenotypes, using linear regression.

**RESULTS:**

*APOE* ε4 presence showed greater atrophy by 25.1 mm^3^ in the left hippocampus (*β*: −0.115 standard deviations, 95% confidence interval [CI] [−0.192, −0.039] and 187.7 mm^3^ in frontal pole gray matter (*β*: −0.112, 95% CI [–0.186, −0.039]) (*q *< 0.05). Unfavorable lifestyle accelerated left hippocampal atrophy, and smoking increased white matter hyperintensity volumes (*q *> 0.05). No interactions were observed.

**DISCUSSION:**

Our study reinforces the independent effect of *APOE* ε4 on longitudinal brain atrophy. Lifestyle may help preserve structural brain health, and our findings provide no evidence this varies by *APOE* ε4 genotype.

## Highlights

- This longitudinal study included 3265 UK Biobank participants with *APOE* ε4 genotype, multiple daily lifestyle factors, and repeated brain MRI data.
- Among cognitively healthy middle‐aged and older adults, *APOE* ε4 presence showed greater atrophy by 25.1 mm^3^ in the left hippocampus and 187.7 mm^3^ in frontal pole gray matter, compared with ε4 absence, over mean follow‐up of 2.6 years.
- There were nominal associations of overall unfavorable lifestyle with greater left hippocampal atrophy and smoking with increased WMHs.
- Lifestyle may help preserve structural brain health, and our findings suggest that these benefits did not significantly vary by *APOE* ε4 status.

## 1 BACKGROUND

With longer life expectancy, age‐related neurodegenerative diseases, such as dementia, affected 57 million people worldwide in 2021 and was the seventh leading cause of death and one of the major causes of disability and dependency.^1^, ^2^, ^3^ As a global public health issue, the incidence of dementia is rising, with 10 million new cases diagnosed annually.^2^ Recent pharmacological interventions for Alzheimer's disease (AD), such as lecanemab and donanemab, provide relatively moderate benefit on average, but they are very expensive and not broadly available,^4^ which is why prevention remains a significant priority. Around 45% of dementia cases might be prevented by implementing interventions through lifestyle change, for example, smoking, excessive alcohol consumption, obesity, and physical inactivity.^1^ Hence, lifestyle could be an important target for intervention to slow brain aging, with volumetric measurements quantified by structural magnetic resonance imaging (MRI).^5^

Brain structure is a logical mediator of cognitive health.^6^ Several risk factors have been found to be associated with brain health through volumetric measures, for example, smoking with smaller gray matter (GM) volumes^7^ and excessive drinking with smaller GM volumes and poorer white matter tract integrity.^8^ *Apolipoprotein E* gene (*APOE*) ε4 allele is the strongest common risk factor for late‐onset AD; carrier frequency among AD cases is approximately 40% to 65%.^3^, ^9^ *APOE* ε4 has well‐established associations with hippocampal atrophy,^10^ as the hippocampal complex is the first site for neurofibrillary tangle development and one of the earliest structures to show volumetric change.^11^ However, these cross‐sectional associations may only have reflected differences in reverse causation or long‐term accumulated effects.^12^ Moreover, while reduced GM volumes in the frontal lobe are associated with declined cognitive performance,^13^ empirical evidence from lifestyle and these structural markers remain unexplored.

Though human cognitive function changes continuously throughout life, preventions in early or midlife might be effective for late‐life brain health.^14^ Given a lack of repeated MRI measurements, few large studies have provided longitudinal evidence regarding lifestyle and brain structural changes: nominal associations of alcohol drinking with thicker cortical thickness of medial orbitofrontal cortex (*N*  = 179),^15^ smoking and white matter hyperintensity (WMH) progression (*N* = 972),^16^ and healthy diet and slower brain structural atrophy (*N* = 1647).^17^ These studies were limited by relatively small sample sizes, overlooked the role of genetic factors, and/or did not account for clusters with other lifestyle behaviors. Robust evidence that healthy lifestyle variables, either alone or in combination, can protect brain health and prevent dementia is still lacking.^18^ Thus, large‐scale cohorts with repeated neuroimaging measurements are important to elucidate the impact of daily lifestyle on longitudinal brain changes over time, while accounting for genetic risk.

To address these limitations, we examined whether and how individual/overall lifestyle variable(s) ‐ smoking, alcohol drinking, diet, sedentary behavior, and physical activity ‐ influenced changes in global brain measures, hippocampal volumes, and regional GM volumes in the frontal lobe, in the context of *APOE* ε4 risk, among people living without dementia.

## 2 METHODS

### 2.1 Study design and participants

Data were obtained from the UK Biobank (UKB), which is a population‐based cohort of 502,412 participants aged 37 to 73 years recruited from 2006 to 2010.^19^ Participants who attended one of 22 assessment centers completed the baseline assessments on physical, lifestyle, sociodemographic, and medical characteristics. In 2014, MRI scanning of the heart, brain, and abdomen for a subgroup of participants began. Between 2014 and 2023, 71,091 participants aged 44 to 85 years had attended the first MRI scan. Participants who reported chronic neurological diseases that could directly affect cognitive function were excluded (Table S1). We focused on lifestyle variables and covariates collected at the first scan as the baseline measures, and brain structural phenotypes at two scans to investigate volumetric changes. This research was completed using the UKB project number 17689.

### 2.2 Lifestyle

#### 2.2.1 Individual lifestyle variables

Participants self‐reported their daily lifestyles at the first scan, including smoking status, alcohol intake, diet types, sedentary behavior duration, and physical activity. Three levels (namely, poor, moderate, and optimal) for each lifestyle were defined based on public health guidelines (Table S2). Smoking was classified as never, former, and current smoker.^7^ Alcohol drinking was defined by type, frequency, and units of weekly alcohol intake and grouped as low, moderate, and high level.^20^ Diet was categorized as healthy, moderate, and unhealthy based on the diet score designated for cardiovascular diseases sharing similar risk factors with dementia.^21^ This score was derived from the food frequency questionnaire collected when participants attended the first MRI scan. The World Health Organization (WHO) recommends limiting the amount of time spent being sedentary.^22^ Using the WHO guidelines for physical activity in populations usually shows a right‐skewed distribution.^22^ Tertiles were therefore used for categories of sedentary behavior duration (time [in hours] spent driving, using a computer, and watching television), and physical activity (weekly metabolic equivalent task units [METs] minutes of walking, moderate and vigorous activity) was defined as low, medium, and high levels.^23^ Intercorrelations between lifestyle variables (*ρ*) ranged from 0.05 to 0.11 (*p *< 0.001) (Table S3).

#### 2.2.2 Lifestyle score and lifestyle categories

**RESEARCH IN CONTEXT**

1. **Systematic review**: The authors reviewed the existing literature on PubMed and Google Scholar. Most studies investigated the associations of individual poor lifestyle and *apolipoprotein E* (*APOE*) ε4 genotype with higher risk of dementia. However, evidence on the relation of daily lifestyles and *APOE* ε4 genotype on brain structural changes, and its interactions among people living without dementia, remains unclear.
2. **Interpretation**: Our findings indicate that *APOE* ε4 shows greater atrophy in the hippocampus and frontal lobe GM over a short‐term follow‐up of 2.6 years. Although only nominal associations were found for specific lifestyle factors (particularly smoking and diet), an overall favorable lifestyle generally helps maintain structural brain health. This beneficial effect appears independent of *APOE* ε4 status.
3. **Future directions**: This work provides a foundation for investigating lifestyle and longitudinal brain volumetric changes considering genetic risk. The findings between lifestyle and brain changes warrant validation in longer follow‐up and larger sample size studies. Moreover, future studies could focus on cross‐ancestry longitudinal studies, which will be needed to clarify genetic effects on brain atrophy.

We assigned zero, one, and two points for each lifestyle variable based on the adherence to poor, moderate, and optimal level of lifestyle behavior, respectively. Two points were assigned for membership of each respective optimal level: never smoking; low‐level drinking; healthy diet; low duration of sedentary behavior; high‐level METs. One point was assigned for each of moderate‐level lifestyles; former smoking, moderate level drinking, moderate diet, medium duration sedentariness, and medium METs; and 0 for poor ones. An unweighted lifestyle score was summed across these five three‐level lifestyle variables, ranging from 0 to 10, with a higher score indicating a healthier lifestyle (i.e., 10 is best, 0 is worst). Based on the frequencies of participants’ lifestyle scores (Figure S1), participants who scored 0, 1, 2, or 3 were classed as an overall unfavorable lifestyle; 4, 5, or 6 as overall moderate lifestyle; 7, 8, 9, or 10 as overall favorable lifestyle.^8^ For example, someone who never smoked and had low risk drinking (two points each), moderate diet (one point), low sedentariness (two points), and high METs (two points) would receive nine points and be included in the favorable lifestyle group. This approach prioritizes clinical relevance and interpretability over statistical convenience.

#### 2.2.3 Imaging data

Brain structural markers (Table S4) at two scans were measured using MRI performed with a 3T Siemens Skyra scanner (Siemens Healthineers) with VD13 software and a 32‐channel head coil.^24^ We used volumetric measures (unadjusted for head size) from T1‐weighted imaging. These included global brain measures: GM, white matter, total brain; hippocampal measures: left hippocampus, right hippocampus, hippocampal asymmetry (right minus left); frontal lobe regional GM volumes derived from the Oxford Centre for Functional Magnetic Resonance Imaging of the Brain (FMRIB)’s Automated Segmentation Tool (FAST) with subregions (including the frontal pole; superior frontal gyrus; middle frontal gyrus; inferior frontal gyrus, pars triangularis; inferior frontal gyrus, pars opercularis; frontal medial cortex; frontal orbital cortex; frontal operculum cortex) summed across both hemispheres, respectively at two MRI scans for subsequent analysis. These regions were selected as they have shown association with cognitive deficits.^12^, ^13^ We additionally included the volume of WMH from the T2 fluid‐attenuation inversion recovery (FLAIR) structural scan because of strong association with dementia.^25^ WMH data were log‐transformed due to a long‐tail distribution, and we excluded outliers whose values were greater than 5 standard deviations (SDs) from the sample mean (*n* = 1 at the second scan). To estimate brain atrophy, the primary outcomes were the raw volumetric changes of brain markers between two scans.

#### 2.2.4 Genetic data

UKB genotyping was conducted by Affymetrix using a bespoke BiLEVE Axiom array for ∼50,000 participants and the remaining 450,000 on the Affymetrix UKB Axiom array. All genetic data were quality controlled by UKB.^26^ The *APOE* ε genotype is directly genotyped. The two *APOE* ε single nucleotide polymorphisms – rs7412 and rs429358 – were both in Hardy‐Weinberg equilibrium (*p *> 0.05) assessed with PLINK version 1.90.^27^ For enough granularity to test lifestyle and genetics interactions, a dominant *APOE* ε4 model – ε3ε4 and ε4ε4 versus ε2ε2, ε2ε3, and ε3ε3 – was used because previous research showed that about 2% of the cohort were ε4 homozygotes.^28^ The rare ε2ε4 genotype was removed because it had potentially both risk and protective alleles.^29^

#### 2.2.5 Covariates

We used information from questionnaires completed at the first scan: age, sex (male or female), and Townsend deprivation index.^30^ Genetic and imaging covariates were included: genotypic arrays, the first 10 principal components for stratification, MRI site (*n* = 4), and intracranial volume (ICV), which was generated by summing the volumes of white matter, GM, and ventricular cerebrospinal fluid at the first scan,^31^ and time intervals, which were the interval in days between the dates of attending two scans. Since these measures were ascertained prior to brain imaging, they cannot be mediators and were treated as confounders (associated with both exposures and outcomes).

#### 2.2.6 Statistical analyses

For differences in baseline characteristics (the first scan) of participants across *APOE* ε4 genotype, categorical variables were compared using chi‐squared tests and continuous variables using *t*‐tests or Mann–Whitney U tests, as appropriate.

We examined the relations between lifestyle, *APOE* ε4 genotype, and longitudinal brain atrophy by linear regression. To quantify atrophy, raw volumetric change for each brain marker was calculated by subtracting the volume at the first scan from the second scan (mm^3^). We then calculated the *z*‐score of raw volumetric change for each marker to ensure comparability. In the primary analyses, we examined the associations of lifestyle categories and *APOE* ε4 genotype, respectively, with both raw volumetric changes of brain markers and their *z*‐scores. The raw change models were adjusted for corresponding baseline brain markers, while the *z*‐score change models were adjusted for the according their baseline *z*‐score. All models were further controlled for sociodemographic factors (age at first scan, sex, deprivation index), time intervals, and genetic‐ and imaging‐related factors (genotypic arrays, MRI site, the first 10 principal components, ICV). In the secondary analyses, to evaluate the independent contribution of individual lifestyle, we modeled each variable individually and subsequently included them in a mutually adjusted model. Moreover, interactions between *APOE* ε4 genotype with lifestyle categories and individual lifestyle variables on brain structural atrophy were tested. We further performed interaction analyses between sex × *APOE* ε4 genotype among relatively younger (≤60 years) and older (>60 years) participants on brain atrophy. We additionally tested association between continuous lifestyle scores and brain structural changes.

Both standardized *β* and unstandardized *β* were reported. Positive standardized *β*s indicate that the exposure group had less volume loss (i.e., attenuated atrophy) and are therefore “better” compared to the reference group, whereas negative *β*s indicate greater volume loss (i.e., accelerated atrophy). This direction is reversed for WMH volumes (Figure S2). The unstandardized *β*s represent the additional mean difference in absolute volumetric change (measured in mm^3^) between the exposure and reference groups over the follow‐up period, providing a direct interpretation of the magnitude of brain structural changes.

Analyses were conducted in Stata version 18 and R (version 4.2.3). We report two‐sided *p* values and Benjamini–Hochberg adjusted *q* values, where a threshold of 0.05 was applied. For associated factors (*p *< 0.05), predictive margins were used to estimate the adjusted mean differences in brain volumetric changes across groups.

## 3 RESULTS

### 3.1 Baseline characteristics

**FIGURE 1:**
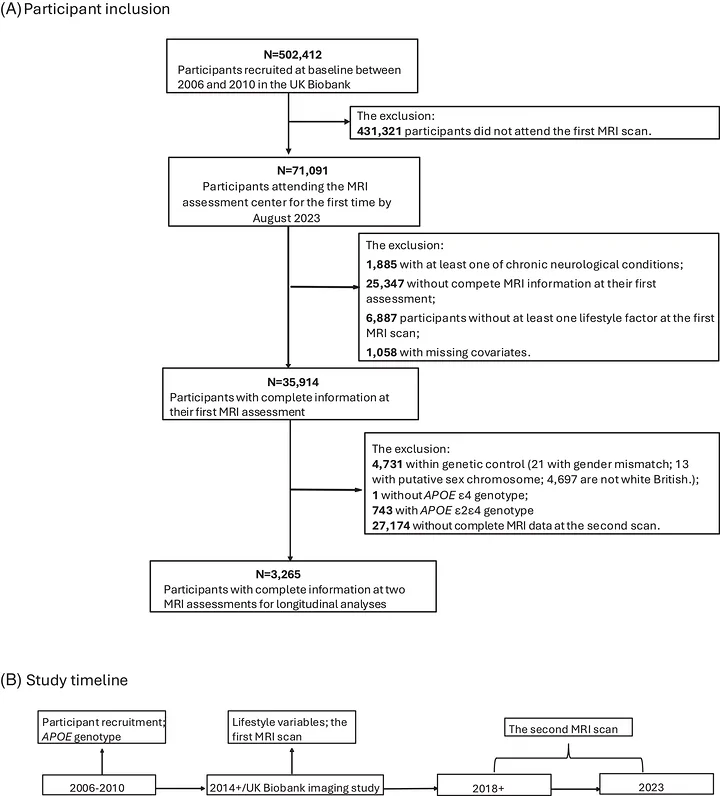
Flowchart (A) and timeline (B) summarizing participant selection and inclusion processes, including the reasons excluding participants.

A total of 3265 cognitive healthy participants were included with complete information on lifestyle factors, *APOE* ε4 genotype, covariates at the first scan, and brain structural volumes at two scans (Figure 1). The mean of follow‐up was 2.6 years. The 3265 participants had a mean (SD) age of 61.5 (7.4) years at the first scan, and 1678 (51.4%) were female (Table 1). There were 809 (24.8%) participants carrying at least one copy of the ε4 allele, who were on average younger, had more favorable lifestyle, had smaller middle frontal gyrus GM, larger inferior frontal gyrus and larger pars triangularis GM. There were 351 (10.8%) participants with unfavorable lifestyles who had the largest volume of WMH with the median (IQR) of 2818 (4371) mm^3^ in Table S5.

**TABLE 1:** Characteristics of participants at baseline MRI assessment (*N* = 3265).

	Total	*APOE* ε4 absence	*APOE* ε4 presence
*N*	3265	2456	809
**Age, years (mean [SD])**	61.5/7.4	61.7/7.4	60.8/7.3
**Sex (*n*/%)**			
Female	1678/51.4	1240/50.5	438/54.1
Male	1587/48.6	1216/49.5	371/45.9
**Deprivation index quantile (*n*/%)**			
Q1 (least deprived)	656/20.1	487/19.8	169/20.9
Q2	650/19.9	477/19.4	173/21.4
Q3	653/20.0	507/20.6	146/18.1
Q4	653/20.0	492/20.0	161/19.9
Q5 (most deprived)	653/20.0	493/20.1	160/19.8
**Lifestyle factors (*n*/%)**			
Smoking status			
Current smoker	106/3.3	77/3.1	29/3.6
Former smoker	1015/31.1	764/31.1	251/31.0
Never	2144/65.7	1615/65.8	529/65.4
**Alcohol consumption**			
High risk	980/30.0	760/30.9	220/27.2
Moderate risk	957/29.3	707/28.8	250/30.9
Low risk	1328/40.7	989/40.3	339/41.9
**Diet**			
Unhealthy diet	673/20.6	496/20.2	177/21.9
Moderate diet	2217/67.9	1691/68.9	526/65.0
Healthy diet	375/11.5	269/11.0	106/13.1
**Sedentary behavior**			
High duration (6.5 to 18 h)	812/24.9	613/25.0	199/24.6
Medium duration (4.5 to 6 h)	1197/36.7	919/37.4	278/34.4
Low duration (0 to 4 h)	1256/38.5	924/37.6	332/41.0
**Physical activity**			
Low METs (0 to 1466)	1093/33.5	819/33.4	274/33.9
Medium METs (1470 to 3279)	1084/33.2	826/33.6	258/31.9
High METs (3285 to 19,278)	1088/33.3	811/33.0	277/34.2
**Lifestyle levels (*n*/%)**			
Unfavorable lifestyles (0 to 3 points)	351/10.8	264/10.7	87/10.8
Moderate lifestyles (4 to 6 points)	1751/53.6	1347/54.9	404/49.9
Favorable lifestyles (7 to 10 points)	1163/35.6	845/34.4	318/39.3
**Lifestyle score (mean [SD]) (points)**	5.8/1.8	5.8/1.8	5.8/1.8
**Follow‐up duration, years, mean (SD)**	2.6/1.1	2.6/1.1	2.6/1.1
**Brain MRI volumes (mean [SD]) (cm^3^)**			
Gray matter	625.93/55.17	626.17/55.55	625.19/54.03
White matter	551.81/61.61	552.88/62.00	548.56/60.31
Total brain	1177.74/111.23	1179.05/112.02	1173.75/108.76
White matter hyperintensities, median (IQR)	2.62/3.48	2.64/3.46	2.48/3.55
Left hippocampus	3.84/0.48	3.84/0.48	3.82/0.45
Right hippocampus	3.96/0.49	3.96/0.49	3.96/0.50
Hippocampal asymmetry	0.13/0.42	0.12/0.42	0.14/0.43
Frontal pole GM	50.17/5.62	50.20/5.56	50.06/5.82
Superior frontal gyrus GM	21.01/3.18	21.01/3.17	21.00/3.21
Middle frontal gyrus GM	20.00/3.27	20.06/3.22	19.80/3.42
Inferior frontal gyrus, pars triangularis GM	4.79/0.92	4.77/0.91	4.85/0.95
Inferior frontal gyrus, pars opercularis GM	5.21/0.89	5.20/0.88	5.24/0.92
Frontal medial cortex GM	3.83/0.61	3.83/0.60	3.85/0.66
Frontal orbital cortex GM	12.87/1.52	12.88/1.51	12.82/1.56
Frontal operculum cortex GM	2.91/0.45	2.91/0.44	2.93/0.46
Intracranial volume	1212.20/115.03	1213.68/115.73	1207.71/112.83

*Note*: Brain MRI volumes are raw values (uncorrected for head size).

Abbreviations: *APOE*, apolipoprotein E; GM, gray matter; IQR, interquartile range index; MET, metabolic equivalents task unit; MRI, magnetic resonance imaging; SD, standard deviation.

**TABLE 2:** Longitudinal associations between *APOE* ε4 genotype and brain structural changes.

	*APOE* ε4 genotype					
Brain structural phenotypes	Standardized *β*	95% CI	Unstandardized β	SE	*P* value	*q* value
			(in mm^3^)			
**Global measures**						
Volume of gray matter	−0.038	[−0.113, 0.036]	−463.6	460.5	0.314	0.433
Volume of white matter	−0.035	[−0.111, 0.040]	−478.7	521.8	0.359	0.433
Total brain volume	−0.052	[−0.127, 0.022]	−832.8	601.4	0.166	0.311
Volume of white matter hyperintensities	0.005	[−0.074, 0.083]	18.6	161.5	0.908	0.908
**Hippocampal volumes**						
Volume of left hippocampus	**−0.115^†^**	**[−0.192, −0.039]**	**−25.1^†^**	**8.5**	**0.003**	**0.022**
Volume of right hippocampus	**−0.079**	**[−0.154, −0.004]**	**−17.0**	**8.2**	**0.039**	**0.146**
Hippocampal asymmetry	0.038	[−0.037, 0.113]	10.0	10.0	0.318	0.433
**Regional GM volumes in frontal lobe**						
Frontal pole	**−0.112^†^**	**[−0.186, −0.039]**	**−187.7^†^**	**62.5**	**0.003**	**0.022**
Superior frontal gyrus	**−0.088**	**[−0.165, −0.012]**	**−82.4**	**36.5**	**0.024**	**0.120**
Middle frontal gyrus	−0.064	[−0.140, 0.013]	−55.2	33.7	0.102	0.219
Inferior frontal gyrus, pars triangularis	−0.051	[−0.128, 0.025]	−11.5	8.8	0.189	0.315
Inferior frontal gyrus, pars opercularis	−0.066	[−0.142, 0.011]	−14.3	8.5	0.092	0.219
Frontal medial cortex	−0.019	[−0.094, 0.057]	−3.7	7.5	0.626	0.671
Frontal orbital cortex	−0.034	[−0.108, 0.041]	−15.2	17.1	0.375	0.433
Frontal operculum cortex	−0.073	[−0.149, 0.003]	−8.7	4.6	0.061	0.183

*Note*: Bold type indicates *p* < 0.05; † denotes *q* < 0.05 (Benjamini–Hochberg adjusted). Standardized and unstandardized *β*s were estimated by linear regression models, where raw volumetric changes are regressed on *APOE* ε4 status with the reference of absence, adjusted for corresponding baseline brain structural volume, smoking, alcohol drinking, diet, sedentariness, physical activity, age, sex, time intervals, genotypic array, MRI sites, genetic principal components, deprivation index, intracranial volume among 3265 participants, except for the white matter hyperintensity models (*N* = 3264).

Abbreviations: CI, confidence interval; MRI, magnetic resonance imaging; SE, standard error.

### 3.2 Associations between lifestyle, *APOE* ε4 genotype, and longitudinal brain atrophy

#### 3.2.1 Lifestyle categories

**FIGURE 2:**
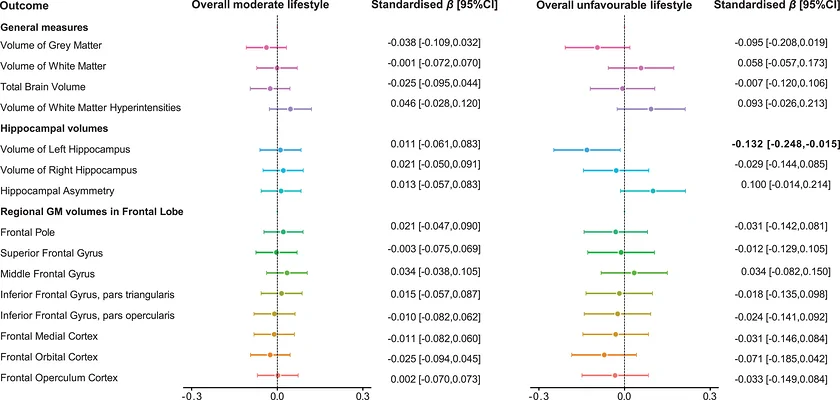
Associations between lifestyle categories and the *z*‐scores of longitudinal changes in brain structural markers. (Bold type indicates *p* < 0.05; † denotes *q* < 0.05, Benjamini–Hochberg adjusted.) Standardized betas were estimated by linear regression models, where the *z*‐scores of raw volumetric changes are regressed on lifestyle categories with the reference of favorable lifestyle, adjusted for corresponding *z*‐score of baseline brain structural volume, *APOE* ε4 genotype, age, sex, genotypic array, magnetic resonance imaging sites, genetic principal components, deprivation index, time intervals, intracranial volume among 3265 participants, except for the white matter hyperintensity models (*N* = 3264). CI, confidence interval.

Compared with participants living with favorable lifestyle who experienced a mean atrophy of 64.4 mm^3^ in the left hippocampus over time, those with unfavorable lifestyle showed additional accelerated average atrophy of 28.6 mm^3^, equivalent to −0.132 (95% confidence interval [CI]: −0.248 to −0.015, *p *= 0.027) lower *z*‐score of raw changes in the left hippocampus in Figures 2 and S3 and Table S6. However, it did not survive after correction for multiple testing (*q *= 0.405). Additionally, a one‐point increment in lifestyle score (out of 10 points) was not associated with any examined changes of brain markers in Table S7.

#### 3.2.2 *APOE* ε4 genotype

As shown in Table 2 and Figure S4, no association was observed between *APOE* ε4 presence and global brain measures; ε4 was, however, associated with accelerated atrophy in the hippocampus and frontal lobe GM. Specifically, ε4 absence showed a mean volumetric loss by 60.0 mm^3^ in the left hippocampus and 642.9 mm^3^ in the frontal pole GM; ε4 presence was associated with an additional accelerated atrophy of 25.1 mm^3^ in the left hippocampus, with a standardized *β* (95% CI) of −0.115 (−0.192 to −0.039, *p* = 0.003, *q *= 0.022) and 187.7 mm^3^ in frontal pole GM (−0.112, −0.186 to −0.039, *p *= 0.003, *q *= 0.022). Nominal associations were found between ε4 presence and greater atrophy by 17.0 mm^3^ in right hippocampus and 82.4 mm^3^ in superior frontal gyrus, compared with ε4 absence (*q *> 0.05).

#### 3.2.3 Individual lifestyle variables

**FIGURE 3:**
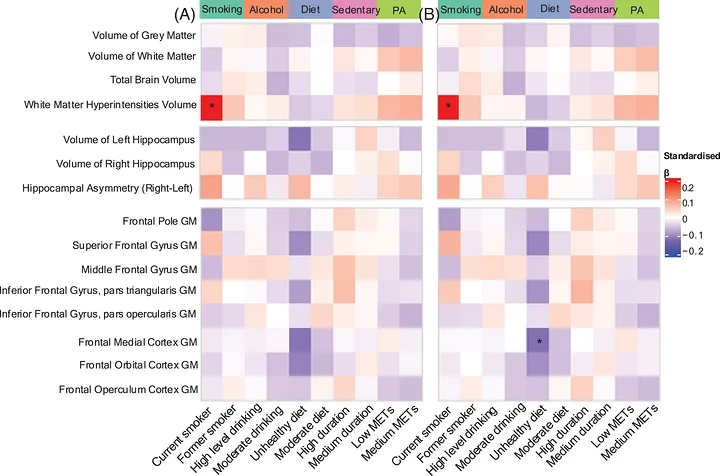
Associations of each lifestyle variable individually (A) and simultaneously (B) modeled on *z*‐scores of longitudinal changes in brain structural markers. Standardized betas were estimated by linear regression models, where *z*‐scores of raw volumetric changes are regressed on each individual lifestyle individually (A), then an overall lifestyle regressed on brain metrics simultaneously (B) among 3265 participants, with the reference of each optimal lifestyle. All models were adjusted for corresponding *z*‐score of baseline brain structural volume, *APOE* ε4 genotype, age, sex, genotypic array, magnetic resonance imaging sites, genetic principal components, deprivation index, time intervals, intracranial volume among 3265 participants, except for the white matter hyperintensity models (*N* = 3264). Stronger associations are indicated by darker shades, light/dark red indicates a positive association, light/dark purple indicates a negative association. MET, metabolic equivalent task units (in minutes); PA, physical activity. *p < 0.05; †*q* < 0.05 (Benjamini–Hochberg adjusted).

In Figure 3 and Tables S8 and S9, no association was observed between alcohol consumption, sedentary behavior, or physical activity and longitudinal structural changes (*p* > 0.05) in either model. However, several nominally significant associations attenuated to non‐significance when corrected for multiple comparisons. For instance, compared with non‐smokers who had a mean progression of 467.3 to 468.9 mm^3^ in Figure S5, current smokers exhibited an additional increase in WMH volume ranging from 793.2 to 809.9 mm^3^ across the individual model (*p *= 0.046, *q *= 0.690) and simultaneous model (*p *= 0.043, *q *= 0.645). We also observed an association between unhealthy diet and decreased frontal medial cortex GM (−0.124, −0.247 to −0.001, *p *= 0.048, *q *= 0.450) in the simultaneous models.

#### 3.2.4 Interactions of lifestyle and *APOE* ε4 genotype on brain structural phenotypes

No interaction was observed between lifestyle categories and *APOE* ε4 genotype in relation to changes across investigated marker (Table S10). Two‐way interaction tests between individual lifestyle variable and *APOE* ε4 genotype yielded no associations in relation to brain structural changes (Table S11). Several nominal interactions were observed between *APOE* ε4 genotype and sex, mostly in older group (Tables S12 and S13), particularly in the frontal lobe GM, but none survived after multiple testing comparison.

## 4 DISCUSSION

In this longitudinal study, we examined the relations hips between daily lifestyle and *APOE* ε4 genotype with brain structural changes over the mean follow‐up of 2.6 years. Compared with ε4 absence, ε4 presence showed greater, accelerated atrophy in the hippocampus and frontal lobe GM. There were nominal associations between unfavorable lifestyle and left hippocampal atrophy, current smokers with increased WMH accumulation, as well as unhealthy diet and frontal medial cortex GM atrophy, but we cannot rule out chance findings. No interactions were found between lifestyle and *APOE* ε4 genotype on longitudinal brain atrophy.

Cognitively healthy participants with the ε4 allele showed significantly greater volumetric loss in the left hippocampus over a mean follow‐up of 2.6 years in our study. Numerous studies have established the associations between *APOE* ε4 and hippocampal atrophy in individuals with AD or mild cognitive impairment.^10^ Carriers may be simultaneously affected by increased amyloid beta and tau pathologies, neuroinflammation, synaptic dysfunction, or metabolism disruptions, where their structural damage is already pronounced.^32^ Thus, it is hard to know the true causal effect of *APOE* ε4 on brain volumetric loss. Our study reveals that ε4 presence exerts differential effects even in a short period of 2.6 years in later life, which highlights the importance of understanding genetic neurodegenerative trajectories. Specifically, we offer valuable insights for clinical trials to evaluate novel disease‐modifying therapies, such as lecanemab and donanemab.^4^

Previous research has mostly focused on changes in total hippocampal volume^33^, ^34^ and overlooked the regionally specific involvement of the hippocampus.^35^ Only one cross‐sectional study focused on bilateral hippocampal reductions, especially in the right hippocampus among 22 young healthy *APOE* ε4 carriers.^36^ Yet our results demonstrated the additional greater loss of 25.1 mm^3^ specifically in the left hippocampus, aligning with the hypothesis that hippocampal dysfunction starts asymmetrically on the left.^35^ Although we used data from a large population with repeated, high‐resolution, automated segmentation of brain volumes to identify the magnitude of hippocampal changes, it remains possible that certain subtle effects require larger cohorts to achieve sufficient statistical power.

Our research suggested that ε4 presence accelerated frontal pole GM atrophy by 187.7 mm^3^. The frontal pole supports high‐level cognitive functions, including analogical reasoning, prospective memory, exploratory decision‐making, and the integration of diverse information.^37^ We also found that ε4 presence was nominally associated with accelerated atrophy in the superior frontal gyrus. Future research should further investigate the mechanisms underlying frontal pole and superior frontal gyrus vulnerability in *APOE* ε4 carriers. Pathological processes of *APOE* ε4 could contribute to neuronal dysfunction and loss, leading to cognitive decline and progressive brain atrophy,^38^ as observed on structural MRI.

Notably, we found a nominal association between unfavorable lifestyle and accelerated atrophy by 28.6 mm^3^ in the left hippocampus. This relatively subtle effect contrasts with the Lancet Commission report suggesting 45% of dementia cases could be prevented through modifiable risk factors.^1^ Though our finding represents a novel contribution in terms of magnitude of lifestyle, it should be interpreted as effectively null pending further replication in larger cohorts. Several factors might have influenced our findings. First, the relatively short follow‐up period between two scans might be insufficient to detect the cumulative structural changes caused by lifestyle. Second, calculating raw volumetric changes could increase the measurement error (although we did control for baseline values). The smaller sample size compared with cross‐sectional analysis using data from the first scan^8^ only may have limited statistical power to detect small effects. Furthermore, our sample was composed of relatively young and healthy individuals who might have had less brain atrophy, which typically occurs in older people.

No statistically significant modification by *APOE* ε4 was detected in this sample. This implies that adopting favorable lifestyle may exert comparable neuroprotective effects in both ε4 carriers and non‐carriers. However, given the small number of unfavorable‐lifestyle *APOE* ε4 carriers, the results should be interpreted with caution. Similarly, while a previous study reported a complex interplay between age, *APOE* ε4 genotype, and sex,^39^ nominal interactions were observed in our study. The current sample of over 3000 participants provides sufficient power for establishing main effects, but it remains underpowered for detecting complex interaction terms. Future studies with larger sample sizes and longer follow‐up times are needed to evaluate these interactions with more granularity and statistical power.

Multiple lines of significant cross‐sectional evidence on individual daily lifestyle with smaller brain volumes have been reported,^7^, ^8^, ^40^, ^41^ including our prior work.^8^ However, cross‐sectional designs captured only a single time point and may miss early or subtle neurodegenerative changes.^12^ The current study explicitly extends our previous research into a longitudinal subsample with repeated MRI, though showing mostly null associations between lifestyle and brain atrophy. This aligns with another longitudinal study (*N* = 179) demonstrating no associations between lifestyles and changes in cortical thickness brain structure.^15^ The longitudinal atrophy may explain the substantial heterogeneity observed in cross‐sectional findings. We found that current smokers were nominally associated with increased volumes of WMH by approximately 800 mm^3^, consistent with previous study, though without stating the magnitude,^16^ compared with non‐smokers. This requires further replication in larger cohorts. Chronic exposure to noxious agents in cigarette smoking may directly compromise neuronal and cellular membrane functions of cerebral tissue and lead to cardiovascular and cerebrovascular disease.^42^ Moreover, WMHs, which reflect white matter injury visible on MRI, have been linked to worse cognitive impairment and increased risk of cerebrovascular diseases and AD.^43^

This study's major strengths were the relatively large sample size with repeated brain MRI data for conducting longitudinal analyses and the simultaneous inclusion of multiple daily lifestyle variables that allowed us to investigate their individual and combined effects on brain atrophy. The use of raw volumetric changes, adjusted for corresponding baseline brain phenotypes, yielded intuitive and robust results. To assess the comprehensive impact of lifestyle on brain structural atrophy, we classified participants into three categories based on the characteristics of lifestyle behaviors. This approach allowed us to explore risk stratification and potential intervention strategies on a gradient of lifestyle profiles, rather than the number of widely used variables showing adherence to a healthy lifestyle,^44^, ^45^ which might overlook the relation among participants with intermediate lifestyles.

The study also had limitations. First, healthy volunteer bias may have influenced our findings. Participants who returned for repeat imaging were likely healthier and more engaged than the broader UKB imaging cohort,^46^ which could have influenced the observed associations. Second, there was likely survival bias. As individuals at higher genetic risk, more healthy and resilient ε4 carriers who were able to attend multiple UKB assessments might have been overrepresented, potentially further influencing the true associations between *APOE* ε4 and brain structural decline. Third, despite the fact that participants with at least one neurological condition were excluded, those with cognitive impairments were not specifically identified. Thus, the results might have been overestimated. Fourth, our sample was restricted to predominantly White British participants from the UKB to minimize population stratification, which may limit global generalizability. A previous study reported that the risk associated with *APOE* ε4 allele varied by ancestry,^47^ so future cross‐ancestry longitudinal studies are needed to clarify the genetic effects on brain atrophy. Finally, despite the longitudinal design and the adjustment of a comprehensive list of confounders, residual confounding cannot be ruled out.

In conclusion, we found that *APOE* ε4 caused accelerated atrophy in the hippocampus and frontal lobe GM, even in a short period among healthy individuals, whereas lifestyle could help preserve structural brain health and suggests that these benefits did not significantly vary by *APOE* ε4 status. These exploratory findings between lifestyle and brain changes warrant validation in longer follow‐up studies.

## CONFLICT OF INTEREST STATEMENT

The authors declare no conflicts of interest. Author disclosures are available in the Supporting Information.

## CONSENT STATEMENT

All human subjects provided their fully informed consent.

## Supporting information




Supporting Information

Supporting Information

## Data Availability

Data within this article can be requested from UK Biobank, found here: https://www.ukbiobank.ac.uk.
