# Supplementary material for: Associations between lifestyle, genetic risk for Alzheimer's disease, and longitudinal brain atrophy in the UK Biobank (N = 3265)
Source: Alzheimers Dement (Amst). 2026 Aug 31;18(3):e70470. doi: 10.1002/dad2.70470 (PMC13529823; doi:10.1002/dad2.70470)
Supplement: Supplementary file 1 [file DAD2-18-e70470-s001.pdf]

Associations between lifestyle, genetic risk for Alzheimer's disease, and longitudinal brain atrophy in UK Biobank (N=3265)

Supplementary materials

Figure S1. Histogram showing the distribution of baseline lifestyle scores among 3265 participants

Figure S2. Differences in brain volumetric changes across lifestyle categories

Figure S3. Predictive margins of raw volumetric change in left hippocampus across by lifestyle categories

Figure S4. Predictive margins of raw volumetric change in brain markers stratified across *APOE* ε4 genotype

Figure S5. Predictive margins of raw volumetric change in white matter hyperintensities across smoking status

Table S1. Self-reported diseases exclusion

Table S2. Lifestyle variables definitions

Table S3. Intercorrelations of lifestyle variables at the initial MRI assessment

Table S4. Variables included in brain structural markers

Table S5. Baseline brain structural volumes stratified by lifestyle levels at the initial MRI assessment

Table S6. Longitudinal associations between lifestyle categories and brain structural changes

Table S7. Longitudinal associations between lifestyle score and brain structural changes

Table S8. Longitudinal associations of single lifestyle variable individually modelled on brain structural changes

Table S9. Longitudinal associations of single lifestyle variable simultaneously modelled on brain structural changes

Table S10. Two-way interactions of lifestyle levels × *APOE* ε4 genotype on longitudinal brain structural changes

Table S11. Two-way interactions of each lifestyle variable × *APOE* ε4 genotype on longitudinal brain structural changes

Table S12. Two-way interactions of sex × *APOE* ε4 genotype on longitudinal brain structural changes among participants aged ≤ 60 years

Table S13. Two-way interactions of sex × *APOE* ε4 genotype on longitudinal brain structural changes among participants aged > 60 years old

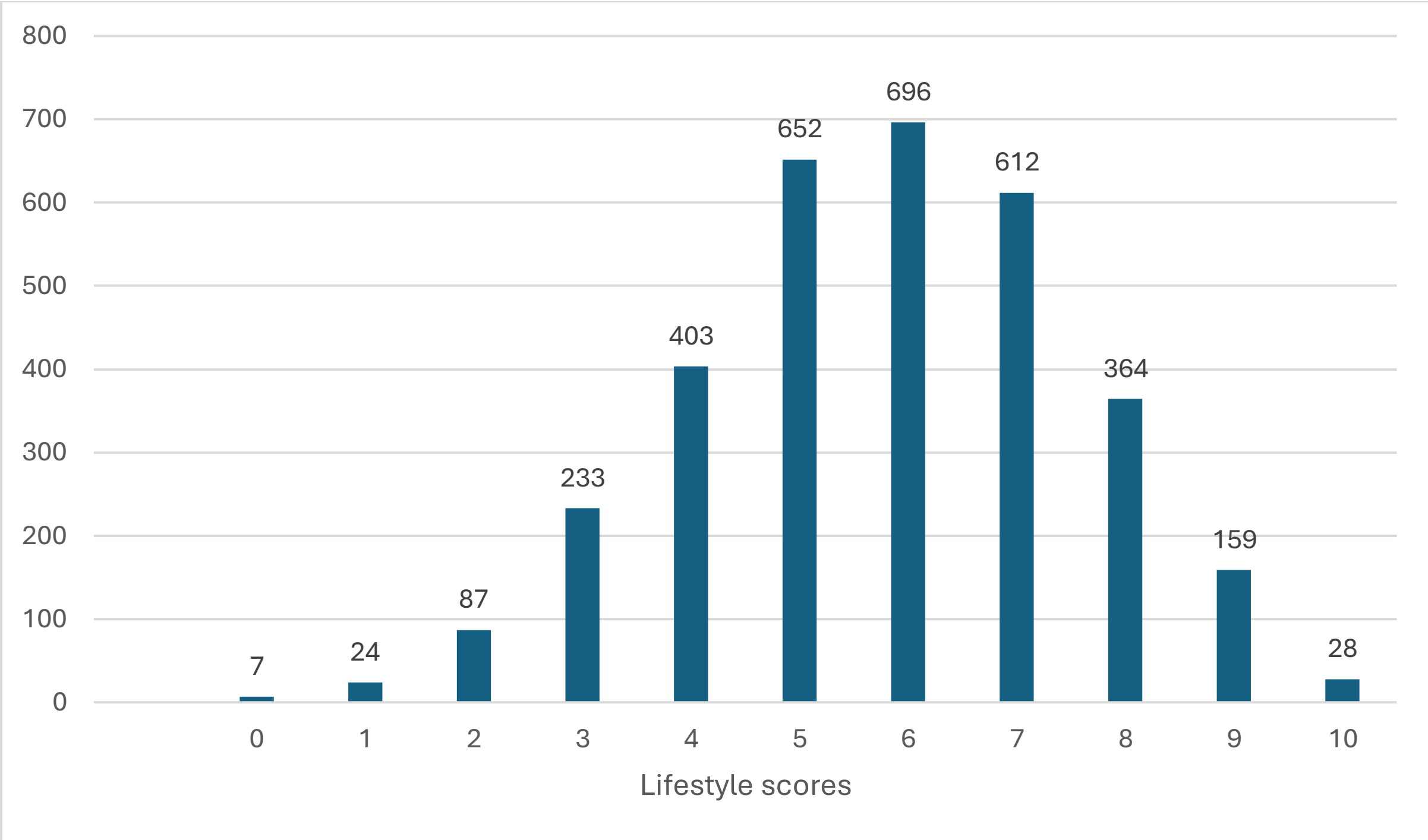

Figure S1. Histogram showing the distribution of baseline lifestyle scores among 3265 participants

Note: Participants who scored 0, 1, 2 or 3 were classed as unfavourable lifestyle; 4, 5, or 6 as moderate lifestyle; 7, 8, 9, or 10 as favourable lifestyle at the baseline MRI scan.

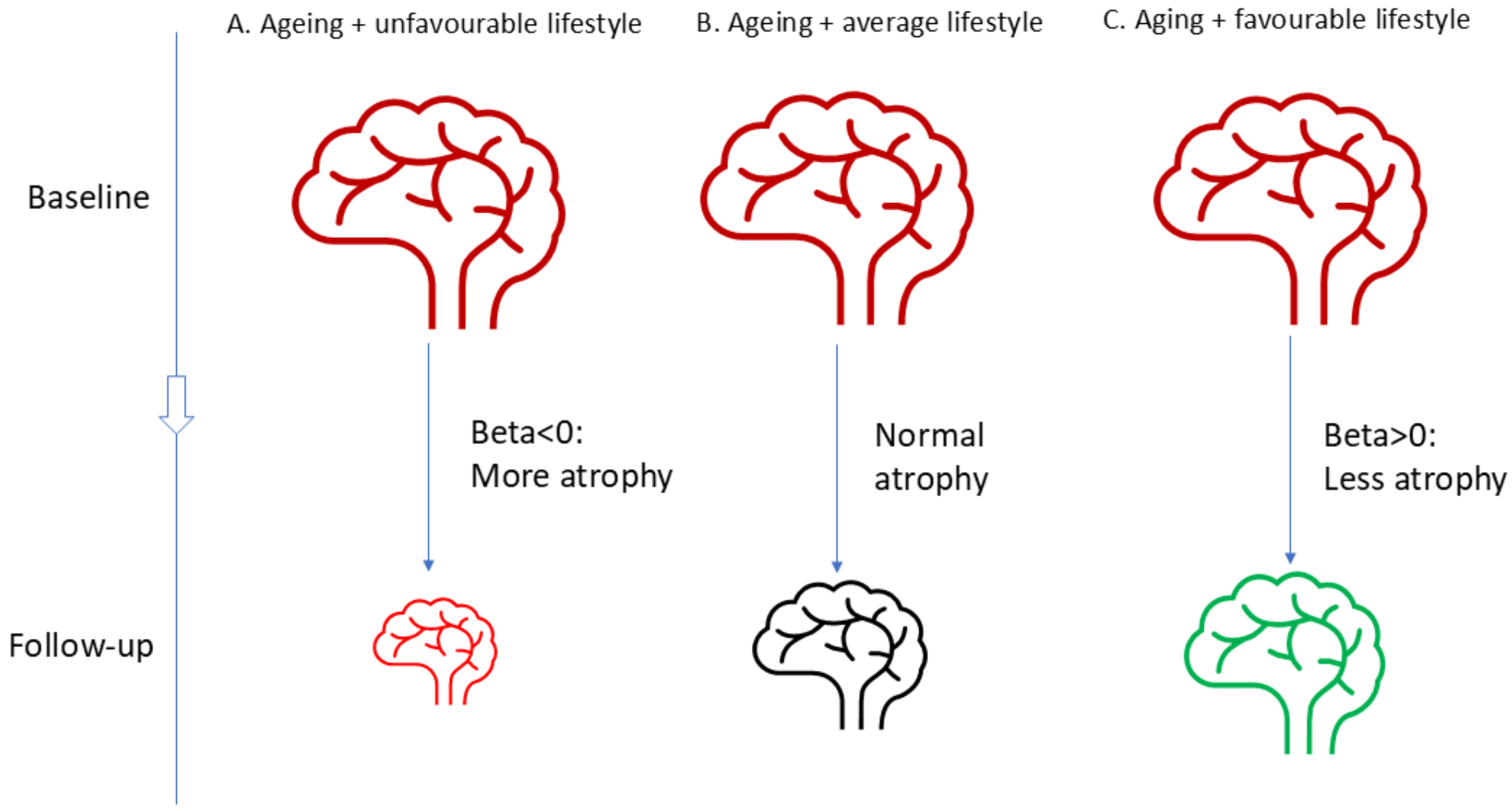

Figure S2. Differences in brain volumetric changes across lifestyle categories

Note: Standardised βs > 0 indicates attenuated atrophy (less volume loss) and accelerated accumulation for white matter hyperintensity volumes, whereas standardised βs < 0 indicates accelerated atrophy (greater volume loss), and attenuated loss for white matter hyperintensity volumes.

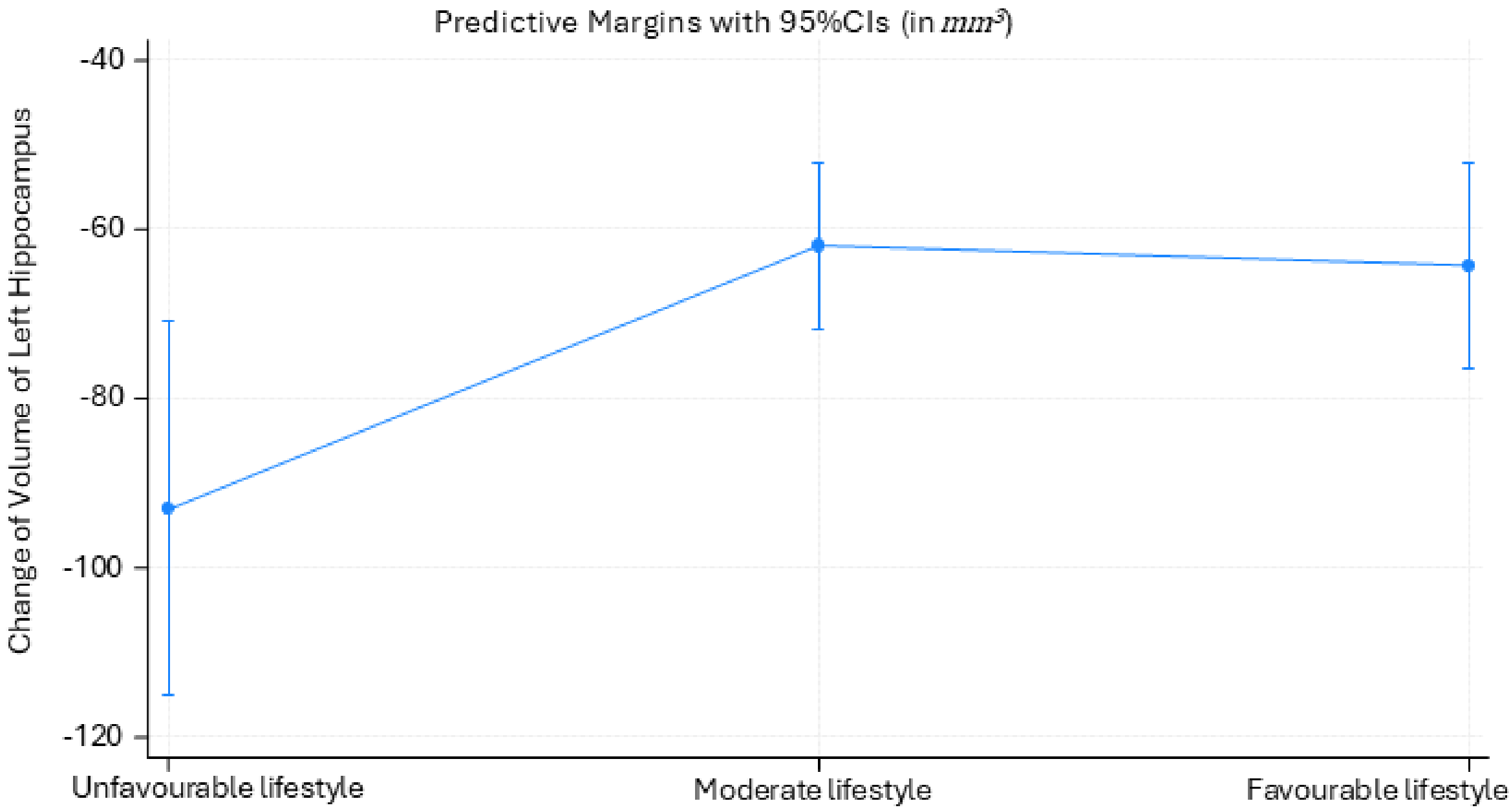

Figure S3. Predictive margins of raw volumetric change in left hippocampus stratified by lifestyle categories

Note: † denotes indicates $q < 0.05$ (Benjamini-Hochberg adjusted). Unstandardised βs and 95% CIs were calculated using linear regression models (in mm^3) on raw volumetric changes in left hippocampus, with the reference of favourable lifestyle, adjusted for corresponding baseline volume of left hippocampus, *APOE* ε4 genotype, age, sex, genotypic array, MRI sites, genetic principal components, deprivation index, time intervals, intracranial volume among 3265 participants. CI, confidence interval.

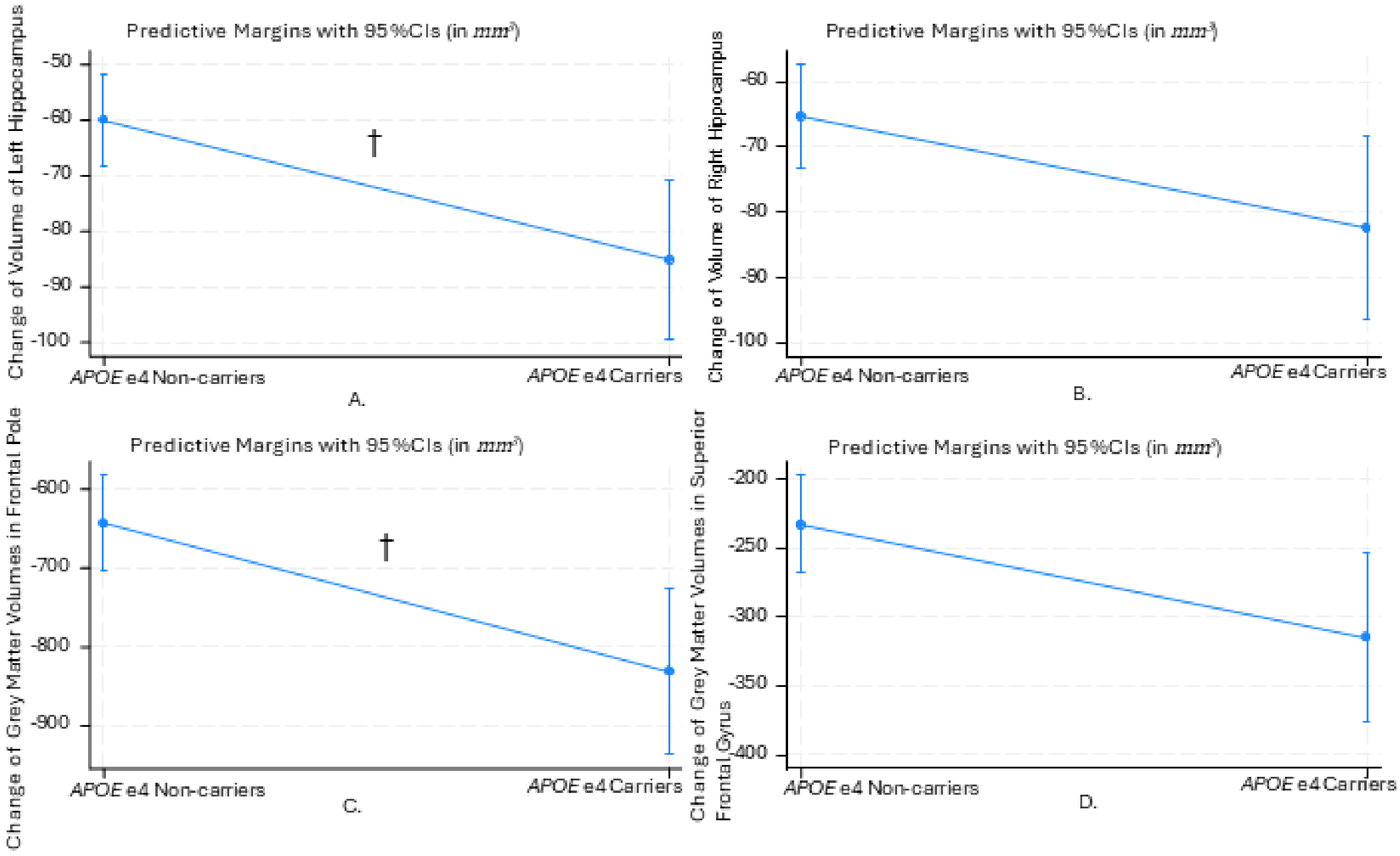

Figure S4. Predictive margins of raw volumetric change in brain markers stratified across *APOE* ε4 genotype

Note: † denotes indicates $q < 0.05$ (Benjamini-Hochberg adjusted). Unstandardised βs and 95% CIs were calculated using linear regression models (in mm^3) on raw volumetric changes in left hippocampus (A), right hippocampus (B), grey matter in frontal pole (C), grey matter in superior frontal gyrus (D), with the reference of *APOE* ε4 absence, adjusted for corresponding baseline brain volume, smoking, alcohol drinking, diet, sedentariness, physical activity, age, sex, genotypic array, MRI sites, genetic principal components, deprivation index, time intervals, intracranial volume among 3265 participants. CI, confidence interval.

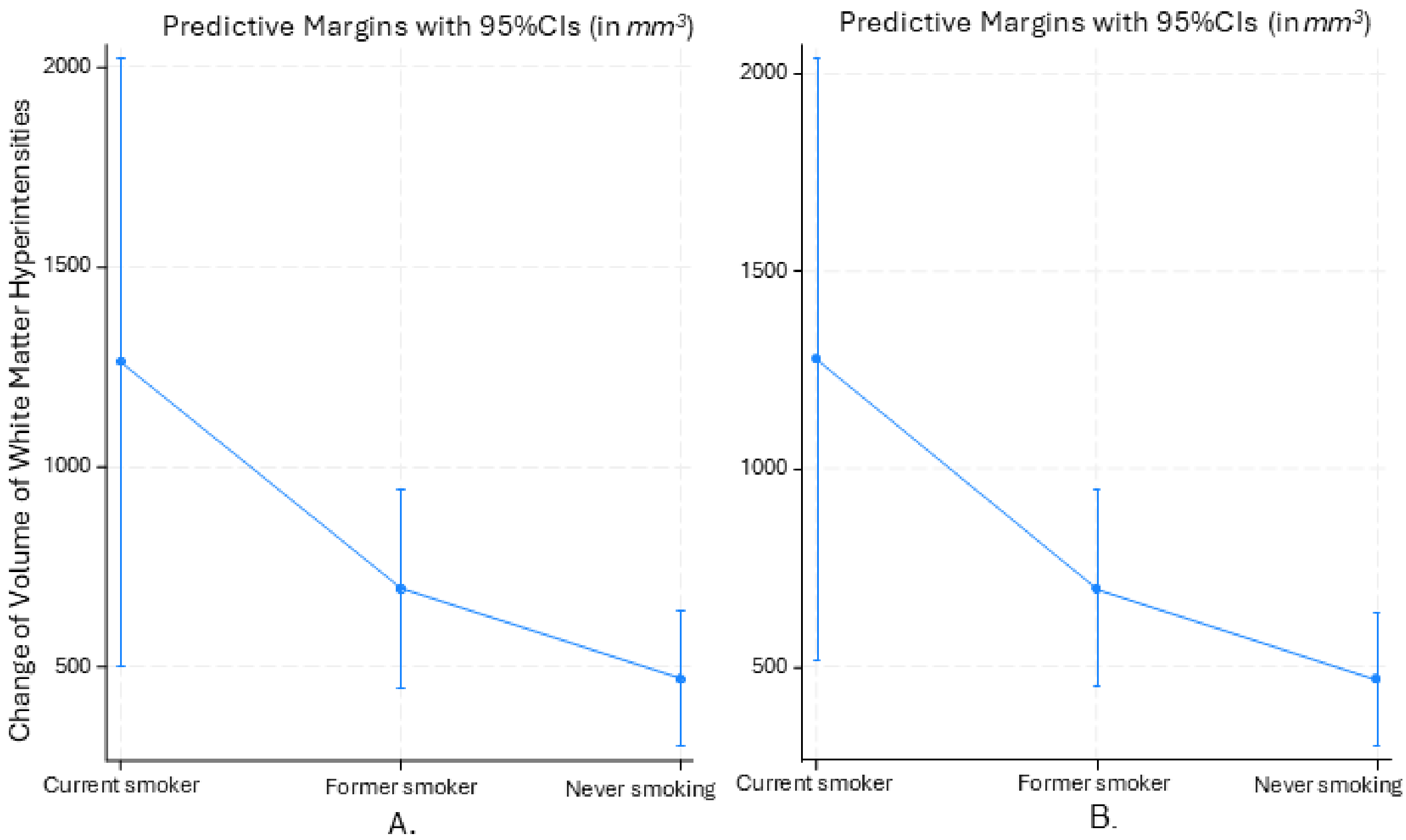

Figure S5. Predictive margins of raw volumetric change in white matter hyperintensities across smoking status

Note: † denotes indicates $q < 0.05$ (Benjamini-Hochberg adjusted). Unstandardised βs and 95% CIs were calculated using linear regression models (in mm^3) on raw changes of volume of white matter hyperintensities without adjustment of other lifestyles (A), and with adjustment of other lifestyles (B), with the reference of never smoking, fully adjusted for corresponding baseline log-transformed volume of white matter hyperintensities, age, sex, genotypic array, MRI sites, genetic principal components, deprivation index, time intervals, intracranial volume among 3264 participants. CI, confidence interval.

Table S1. Self-reported diseases exclusion

**Self-reported diseases**
Brain cancer/primary malignant tumour
Brain haemorrhage
Brain/intracranial abscess
Cerebral aneurysm
Cerebral palsy
Chronic/degenerative neurological problem
Dementia/Alzheimer's disease/cognitive impairment
Encephalitis
Epilepsy
Head injury
Infection of nervous system
Ischaemic stroke
Meningeal cancer/malignant meningioma
Meningioma (benign)
Meningitis
Motor neurone disease
Multiple sclerosis
Neurological injury/trauma
Neuroma (benign)
Other demyelinating condition
Other neurological problem
Parkinson's disease
Spina bifida
Stroke
Subarachnoid haemorrhage
Subdural haematoma
Transient ischaemic attack

Table S2. Lifestyle variables definitions

Lifestyle variable	Field ID	Description	Source and definition
Smoking status	20116	Smoking status	UK Biobank Touchscreen questionnaire at the initial magnetic resonance imaging (MRI) assessment. Smoking was defined as current, former, never smoker.
Alcohol intake	1558	Alcohol intake frequency	UK Biobank Touchscreen questionnaire at the initial MRI assessment. Alcohol intake was calculated by alcohol intake frequency; average weekly red wine intake; average weekly champagne plus white wine intake; average weekly beer plus cider intake; average weekly spirits intake; average weekly fortified wine intake based on UK alcohol units guideline. Alcohol intake was defined here as low, moderate, and high levels, being defined by types, frequencies and units of weekly alcohol intake and grouped based on UK national guidelines: 0-14 units as low risk; 15-35 units for females or 15-50 units for males as moderate risk; > 35 units for females and > 50 units for males as high risk of diseases.
	1568	Average weekly red wine intake	
	1578	Average weekly champagne plus white wine intake	
	1588	Average weekly beer plus cider intake	
	1598	Average weekly spirits intake	
	1608	Average weekly fortified wine intake	
Diet	1309	Fresh fruit intake	UK Biobank Touchscreen questionnaire at the initial MRI assessment. The diet score was derived from the food frequency questionnaire and constructed by the unweighted sum of adherence to the UK dietary guidelines, including: processed meat, red meat individually less than twice per week, total fish more than twice per week, consumption of semi- and skimmed, soya milk, no spread intake, more than five bowls of cereal intake, no salt added to food, more than six glasses of water and more than five servings per day of fruits and vegetables. It ranged from 0 to 9, with higher scores indicating a healthier diet. Participants were categorised into healthy (7–9 points), moderate (4–6 points), and unhealthy (0–3 points).
	1319	Dried fruit intake	
	1299	Salad/raw vegetable intake	
	1289	Cooked vegetable intake	
	1329	Oily fish intake	
	1339	Non-oily fish intake	
	1349	Processed meat intake	
	1389	Pork intake	
	1379	Lamb/mutton intake	
	1369	Beef intake	
	1428	Spread type	
	1458	Cereal intake	
	1478	Salt added to food	
	1528	Water intake	
	1418	Milk type used	
Sedentary behaviour	1090	Time spent driving (in hours)	UK Biobank Touchscreen questionnaire at the initial MRI assessment. Sedentary behaviour was calculated by summing the time spent driving, using computer, and watching television in a typical day.
	1080	Time spent using computer (in hours)	
	1070	Time spent watching television (in hours)	
Physical activity	22038	MET minutes per week for moderate activity	UK Biobank Touchscreen questionnaire at the initial MRI assessment. Physical activity was calculated by metabolic equivalents of task (MET) units minutes per week for moderate activity; vigorous activity; walking.
	22039	MET minutes per week for vigorous activity	
	22037	MET minutes per week for walking	

Table S3. Intercorrelations of lifestyle variables at the initial MRI assessment

Spearman's rank correlation coefficients	Smoking status	Alcohol drinking	Diet	Sedentary behaviour	Physical activity
Smoking status	1				
Alcohol drinking	**0.0482**	1			
Diet	**0.0796**	**0.0803**	1		
Sedentary behaviour	**0.0866**	**0.0839**	**0.1135**	1	
Physical activity	-0.0179	-0.0066	**0.0772**	**0.0747**	1

Note: Bold type indicates significant *p* values (*p* <0.001).

Table S4. Variables included in brain structural markers

Field ID	Description
25004	Volume of ventricular cerebrospinal fluid
25006	Volume of grey matter
25008	Volume of white matter
25010	Volume of brain, grey + white matter
25781	Total volume of white matter hyperintensities (from T1 and T2_FLAIR images)
25019	Volume of hippocampus (left)
25020	Volume of hippocampus (right)
25782	Volume of grey matter in Frontal Pole (left)
25783	Volume of grey matter in Frontal Pole (right)
25786	Volume of grey matter in Superior Frontal Gyrus (left)
25787	Volume of grey matter in Superior Frontal Gyrus (right)
25788	Volume of grey matter in Middle Frontal Gyrus (left)
25789	Volume of grey matter in Middle Frontal Gyrus (right)
25790	Volume of grey matter in Inferior Frontal Gyrus, pars triangularis (left)
25791	Volume of grey matter in Inferior Frontal Gyrus, pars triangularis (right)
25792	Volume of grey matter in Inferior Frontal Gyrus, pars opercularis (left)
25793	Volume of grey matter in Inferior Frontal Gyrus, pars opercularis (right)
25830	Volume of grey matter in Frontal Medial Cortex (left)
25831	Volume of grey matter in Frontal Medial Cortex (right)
25846	Volume of grey matter in Frontal Orbital Cortex (left)
25847	Volume of grey matter in Frontal Orbital Cortex (right)
25862	Volume of grey matter in Frontal Operculum Cortex (left)
25863	Volume of grey matter in Frontal Operculum Cortex (right)

Table S5. Brain structural volumes stratified by lifestyle levels at the initial MRI assessment

Brain structural volumes (Mean [SD], in cm^3)	Unfavourable (n=351)	Moderate (n=1751)	Favourable (n=1163)
**Global measures**			
Volume of Grey Matter	627.30 (55.04)	625.12 (55.88)	626.73 (54.15)
Volume of White Matter	554.87 (56.62)	552.75 (62.25)	549.46 (62.05)
Total Brain Volume	1182.17 (105.69)	1177.87 (112.75)	1176.19 (110.60)
Volume of White Matter Hyperintensities, median (IQR)	2.82(4.37)	2.75(3.88)	2.37(3.03)
**Hippocampal volumes**			
Volume of Left Hippocampus	3.80 (0.46)	3.83 (0.49)	3.85 (0.46)
Volume of Right Hippocampus	3.95 (0.46)	3.97 (0.50)	3.96 (0.49)
Hippocampal Asymmetry	0.16 (0.40)	0.13 (0.43)	0.11 (0.42)
**Regional GM volumes in Frontal Lobe**			
Frontal Pole	50.31 (5.49)	50.07 (5.57)	50.27 (5.74)
Superior Frontal Gyrus	21.35 (3.17)	20.92 (3.20)	21.03 (3.14)
Middle Frontal Gyrus	19.88 (3.03)	20.02 (3.26)	19.99 (3.36)
Inferior Frontal Gyrus, pars triangularis	4.83 (0.89)	4.79 (0.93)	4.77 (0.92)
Inferior Frontal Gyrus, pars opercularis	5.20 (0.86)	5.20 (0.91)	5.23 (0.87)
Frontal Medial Cortex	3.81 (0.61)	3.81 (0.61)	3.87 (0.62)
Frontal Orbital Cortex	12.95 (1.42)	12.83 (1.51)	12.89 (1.57)
Frontal Operculum Cortex	2.89 (0.41)	2.91 (0.45)	2.92 (0.45)

Note: Brain structural volumes are raw values (uncorrected for head size). SD, standard deviation; IQR, interquartile range index; GM, grey matter.

Table S6. Longitudinal associations between lifestyle categories and brain structural changes

Brain structural volumes	Favourable lifestyle	Moderate lifestyle						Unfavourable lifestyle					
		Standardised β	95% CI	Unstandardised β	SE	*p*-value	*q*-value	Standardised β	95% CI	Unstandardised β	SE	*p*-value	*q*-value
**Global measures**													
Volume of Grey Matter	ref.	-0.038	[-0.109,0.032]	-464.2	432.1	0.283	0.978	-0.095	[-0.208,0.019]	-1141.9	700.5	0.103	0.476
Volume of White Matter	ref.	-0.001	[-0.072,0.070]	-13.4	489.0	0.978	0.978	0.058	[-0.057,0.173]	785.3	793.1	0.322	0.805
Total Brain Volume	ref.	-0.025	[-0.095,0.044]	-403.8	563.7	0.474	0.978	-0.007	[-0.120,0.106]	-110.2	914.9	0.904	0.904
Volume of White Matter Hyperintensities	ref.	0.046	[-0.028,0.120]	185.4	152.2	0.223	0.978	0.093	[-0.026,0.213]	376.1	246.2	0.127	0.476
**Hippocampal volumes**													
Volume of Left Hippocampus	ref.	0.011	[-0.061,0.083]	2.3	8.0	0.768	0.978	**-0.132**	**[-0.248, -0.015]**	**-28.6**	**12.9**	**0.027**	0.405
Volume of Right Hippocampus	ref.	0.021	[-0.050,0.091]	4.5	7.7	0.561	0.978	-0.029	[-0.144,0.085]	-6.3	12.5	0.615	0.839
Hippocampal Asymmetry	ref.	0.013	[-0.057,0.083]	3.4	9.4	0.714	0.978	0.100	[-0.014,0.214]	26.1	15.2	0.086	0.476
**Regional GM volumes in Frontal Lobe**													
Frontal Pole	ref.	0.021	[-0.047,0.090]	35.7	58.6	0.542	0.978	-0.031	[-0.142,0.081]	-50.9	95.0	0.592	0.839
Superior Frontal Gyrus	ref.	-0.003	[-0.075,0.069]	-2.7	34.3	0.938	0.978	-0.012	[-0.129,0.105]	-10.9	55.6	0.844	0.904
Middle Frontal Gyrus	ref.	0.034	[-0.038,0.105]	29.1	31.6	0.357	0.978	0.034	[-0.082,0.150]	29.3	51.3	0.567	0.839
Inferior Frontal Gyrus, pars triangularis	ref.	0.015	[-0.057,0.087]	3.3	8.2	0.689	0.978	-0.018	[-0.135,0.098]	-4.1	13.4	0.759	0.876
Inferior Frontal Gyrus, pars opercularis	ref.	-0.010	[-0.082,0.062]	-2.1	8.0	0.788	0.978	-0.024	[-0.141,0.092]	-5.3	12.9	0.682	0.853
Frontal Medial Cortex	ref.	-0.011	[-0.082,0.060]	-2.2	7.1	0.758	0.978	-0.031	[-0.146,0.084]	-6.1	11.4	0.597	0.839
Frontal Orbital Cortex	ref.	-0.025	[-0.094,0.045]	-11.0	16.0	0.492	0.978	-0.071	[-0.185,0.042]	-32.0	26.0	0.218	0.654
Frontal Operculum Cortex	ref.	0.002	[-0.070,0.073]	0.2	4.3	0.962	0.978	-0.033	[-0.149,0.084]	-3.9	7.0	0.583	0.839

Note: Bold type indicates p <0.05; † denotes indicates q < 0.05 (Benjamini–Hochberg adjusted). Unstandardised βs (in mm^3) and standardised βs were estimated by linear regression model, with the reference of favourable lifestyle group, adjusted for corresponding baseline brain structural volume and their z-scores respectively, *APOE* ε4 genotype, age, sex, genotypic array, MRI sites, genetic principal components, deprivation index, time intervals, intracranial volume among 3265 participants, except for the volumetric changes of white matter hyperintensity models (N=3264). CI, confidence interval; SE, standard error; GM, grey matter.

Table S7. Longitudinal associations between lifestyle score and brain structural changes

	Lifestyle score (continuous)					
Brain structural volumes	Standardised β	95% CI	Unstandardised β	SE	*p*-value	*q*-value
**Global measures**						
Volume of Grey Matter	0.014	[-0.004,0.033]	172.3	113.4	0.129	0.402
Volume of White Matter	-0.008	[-0.026,0.011]	-103.4	128.3	0.420	0.821
Total Brain Volume	0.002	[-0.016,0.020]	33.1	148.0	0.823	0.910
Volume of White Matter Hyperintensities	-0.017	[-0.036,0.002]	-68.379	39.936	0.087	0.402
**Hippocampal volumes**						
Volume of Left Hippocampus	0.018	[-0.001,0.037]	4.0	2.1	0.057	0.402
Volume of Right Hippocampus	0.000	[-0.018,0.019]	0.0	2.0	0.990	0.99
Hippocampal Asymmetry	-0.017	[-0.036, 0.001]	4.6	2.5	0.063	0.402
**Regional GM volumes in Frontal Lobe**						
Frontal Pole	0.002	[-0.016,0.020]	2.9	15.4	0.849	0.910
Superior Frontal Gyrus	0.003	[-0.016,0.022]	2.5	9.0	0.778	0.910
Middle Frontal Gyrus	-0.007	[-0.026,0.011]	-6.4	8.3	0.438	0.821
Inferior Frontal Gyrus, pars triangularis	0.003	[-0.016,0.022]	0.7	2.2	0.757	0.910
Inferior Frontal Gyrus, pars opercularis	0.005	[-0.014,0.024]	1.1	2.1	0.605	0.907
Frontal Medial Cortex	0.008	[-0.011,0.026]	1.5	1.9	0.419	0.821
Frontal Orbital Cortex	0.014	[-0.004,0.032]	6.3	4.2	0.134	0.402
Frontal Operculum Cortex	0.005	[-0.014,0.024]	0.6	1.1	0.597	0.907

Note: Bold type indicates p <0.05; † denotes indicates q < 0.05 (Benjamini–Hochberg adjusted). Unstandardised βs ((in mm^3) and standardised βs were estimated by linear regression model, adjusted for corresponding baseline brain structural volume and their z-scores respectively, *APOE* ε4 genotype, age, sex, genotypic array, MRI sites, genetic principal components, deprivation index, time intervals, intracranial volume among 3265 participants, except for the volumetric changes of white matter hyperintensity models (N=3264). CI, confidence interval; SE, standard error; GM, grey matter.

Table S8. Longitudinal associations of single lifestyle variable individually modelled on brain structural changes

**Lifestyle variable**	**Smoking**												
**Brain structural volumes**	**Never smoker**	**Former smoker** Standardised β	95% CI	Unstandardised β	SE	*p*-value	*q*-value	**Current smoker** Standardised β	95% CI	Unstandardised β	SE	*p*-value	*q*-value
**Global measures**													
Volume of Grey Matter	ref.	0.014	[-0.057,0.086]	173.7	439.2	0.692	0.956	-0.011	[-0.195,0.173]	-133.9	1135.8	0.906	0.906
Volume of White Matter	ref.	0.010	[-0.062,0.082]	138.3	496.7	0.781	0.956	-0.043	[-0.230,0.144]	-578.1	1284.7	0.653	0.906
Total Brain Volume	ref.	0.026	[-0.044,0.097]	415.4	572.6	0.468	0.956	-0.030	[-0.213,0.153]	-475.3	1480.9	0.748	0.906
Volume of White Matter Hyperintensities	ref.	0.056	[-0.019,0.131]	226.3	153.7	0.141	0.715	**0.196**	**[0.003,0.390]**	**793.2**	**397.897**	**0.046**	0.690
**Hippocampal volumes**													
Volume of Left Hippocampus	ref.	-0.060	[-0.133,0.013]	-13.1	8.1	0.105	0.715	-0.053	[-0.242,0.135]	-11.6	20.9	0.579	0.906
Volume of Right Hippocampus	ref.	-0.053	[-0.125,0.018]	-11.5	7.9	0.143	0.715	0.039	[-0.146,0.224]	8.3	20.3	0.682	0.906
Hippocampal Asymmetry	ref.	0.002	[-0.069,0.073]	0.5	9.5	0.956	0.956	0.093	[-0.092,0.277]	24.2	24.6	0.325	0.906
**Regional GM volumes in Frontal Lobe**													
Frontal Pole	ref.	-0.015	[-0.085,0.055]	-25.6	59.5	0.667	0.956	-0.093	[-0.274,0.088]	-155.3	154.1	0.314	0.906
Superior Frontal Gyrus	ref.	-0.027	[-0.100,0.046]	-25.4	34.8	0.466	0.956	0.063	[-0.126,0.252]	58.8	89.9	0.513	0.906
Middle Frontal Gyrus	ref.	0.039	[-0.033,0.112]	34.1	32.1	0.288	0.956	-0.057	[-0.245,0.131]	-49.2	83.1	0.554	0.906
Inferior Frontal Gyrus, pars triangularis	ref.	-0.003	[-0.076,0.070]	-0.6	8.4	0.942	0.956	0.043	[-0.146,0.232]	9.6	21.6	0.657	0.906
Inferior Frontal Gyrus, pars opercularis	ref.	-0.032	[-0.104,0.041]	-6.9	8.1	0.396	0.956	-0.042	[-0.230,0.147]	-9.1	21.0	0.663	0.906
Frontal Medial Cortex	ref.	-0.010	[-0.082,0.061]	-2.0	7.2	0.775	0.956	-0.018	[-0.203,0.168]	-3.4	18.5	0.853	0.906
Frontal Orbital Cortex	ref.	-0.007	[-0.078,0.064]	-3.2	16.3	0.844	0.956	-0.036	[-0.220,0.147]	-16.3	42.1	0.698	0.906

Frontal Operculum Cortex	ref.	0.016	[-0.056,0.089]	2.0	4.4	0.656	0.956	-0.020	[-0.208,0.168]	-2.4	11.4	0.833	0.906

**Lifestyle variable**	**Alcohol intake**												
	**Low level**	**Moderate level**						**High level**					
**Brain structural volumes**		Standardised *β*	95% CI	Unstandardised *β*	SE	*p*-value	*q*-value	Standardised *β*	95% CI	Unstandardised *β*	SE	*p*-value	*q*-value
**Global measures**													
Volume of Grey Matter	ref.	-0.055	[-0.135,0.026]	-659.0	495.7	0.184	0.597	0.018	[-0.060,0.095]	212.1	480.2	0.659	0.939
Volume of White Matter	ref.	-0.042	[-0.124,0.039]	-571.5	561.5	0.309	0.597	-0.008	[-0.087,0.071]	-107.5	543.4	0.843	0.939
Total Brain Volume	ref.	-0.065	[-0.145,0.014]	-1039.4	647.2	0.108	0.597	0.018	[-0.059,0.095]	285.6	626.7	0.649	0.939
Volume of White Matter Hyperintensities	ref.	0.018	[-0.066,0.103]	74.1	174.2	0.670	0.773	0.009	[-0.073,0.091]	37.1	169.0	0.826	0.939
**Hippocampal volumes**													
Volume of Left Hippocampus	ref.	-0.039	[-0.121,0.044]	-8.5	9.1	0.355	0.597	-0.054	[-0.134,0.026]	-11.8	8.9	0.183	0.939
Volume of Right Hippocampus	ref.	-0.060	[-0.141,0.021]	-13.0	8.9	0.144	0.597	0.003	[-0.075,0.081]	0.6	8.6	0.943	0.943
Hippocampal Asymmetry	ref.	-0.018	[-0.099,0.062]	-4.7	10.8	0.659	0.773	0.053	[-0.025,0.131]	13.8	10.4	0.187	0.939
**Regional GM volumes in Frontal Lobe**													
Frontal Pole	ref.	-0.040	[-0.119,0.039]	-66.6	67.3	0.322	0.597	-0.006	[-0.083,0.070]	-10.5	65.1	0.872	0.939
Superior Frontal Gyrus	ref.	-0.038	[-0.120,0.045]	-35.2	39.3	0.370	0.597	0.009	[-0.071,0.089]	8.7	38.1	0.820	0.939
Middle Frontal Gyrus	ref.	0.035	[-0.047,0.117]	30.6	36.3	0.398	0.597	0.041	[-0.038,0.121]	35.8	35.1	0.308	0.939
Inferior Frontal Gyrus, pars triangularis	ref.	-0.031	[-0.113,0.052]	-6.9	9.5	0.468	0.638	-0.006	[-0.086,0.074]	-1.4	9.2	0.876	0.939
Inferior Frontal Gyrus, pars opercularis	ref.	-0.003	[-0.085,0.080]	-0.6	9.2	0.952	0.952	0.026	[-0.054,0.106]	5.7	8.9	0.522	0.939
Frontal Medial Cortex	ref.	-0.009	[-0.090,0.072]	-1.7	8.1	0.835	0.895	-0.014	[-0.093,0.064]	-2.8	7.8	0.723	0.939
Frontal Orbital Cortex	ref.	-0.052	[-0.133,0.028]	-23.5	18.4	0.201	0.597	-0.022	[-0.100,0.055]	-10.0	17.8	0.574	0.939
Frontal Operculum Cortex	ref.	-0.045	[-0.127,0.037]	-5.3	5.0	0.285	0.597	-0.006	[-0.086,0.073]	-0.8	4.8	0.874	0.939

Lifestyle variable	Diet												
	Healthy diet	Moderate diet						Unhealthy diet					
Brain structural volumes		Standardised β	95% CI	Unstandardised β	SE	*p*-value	*q*-value	Standardised β	95% CI	Unstandardised β	SE	*p*-value	*q*-value
**Global measures**													
Volume of Grey Matter	ref.	0.006	[-0.097,0.109]	68.3	634.6	0.914	0.985	-0.051	[-0.172,0.069]	-622.2	740.3	0.401	0.717
Volume of White Matter	ref.	-0.001	[-0.105,0.103]	-13.4	718.5	0.985	0.985	0.011	[-0.111,0.133]	152.0	838.2	0.856	0.856
Total Brain Volume	ref.	0.002	[-0.100,0.104]	36.7	828.2	0.965	0.985	-0.028	[-0.148,0.091]	-452.1	966.1	0.640	0.749
Volume of White Matter Hyperintensities	ref.	-0.034	[-0.142,0.074]	-139.1	222.5	0.532	0.985	-0.045	[-0.171,0.082]	-180.0	259.9	0.489	0.717
**Hippocampal volumes**													
Volume of Left Hippocampus	ref.	-0.047	[-0.153,0.058]	-10.3	11.7	0.381	0.985	-0.119	[-0.242,0.004]	-25.9	13.6	0.058	0.405
Volume of Right Hippocampus	ref.	-0.062	[-0.165,0.042]	-13.3	11.4	0.243	0.985	-0.039	[-0.160,0.082]	-8.4	13.3	0.526	0.717
Hippocampal Asymmetry		0.004	[-0.100,0.107]	1.0	13.8	0.945	0.985	0.070	[-0.051,0.190]	18.2	16.1	0.256	0.640
**Regional GM volumes in Frontal Lobe**	ref.												
Frontal Pole		0.006	[-0.095,0.107]	9.8	86.0	0.909	0.985	-0.054	[-0.172,0.064]	-89.6	100.4	0.372	0.717
Superior Frontal Gyrus	ref.	-0.029	[-0.135,0.076]	-27.4	50.3	0.586	0.985	-0.097	[-0.220,0.027]	-90.3	58.7	0.124	0.465
Middle Frontal Gyrus	ref.	0.026	[-0.079,0.131]	22.8	46.4	0.623	0.985	-0.024	[-0.147,0.098]	-20.9	54.2	0.699	0.749
Inferior Frontal Gyrus, pars triangularis	ref.	0.015	[-0.090,0.121]	3.4	12.1	0.779	0.985	-0.083	[-0.207,0.040]	-18.8	14.1	0.184	0.552
Inferior Frontal Gyrus, pars opercularis	ref.	0.042	[-0.064,0.147]	9.1	11.7	0.440	0.985	-0.027	[-0.150,0.096]	-5.8	13.7	0.671	0.749
Frontal Medial Cortex	ref.	-0.051	[-0.155,0.052]	-10.1	10.4	0.332	0.985	-0.119	[-0.240,0.002]	-23.3	12.1	0.054	0.405
Frontal Orbital Cortex	ref.	-0.060	[-0.162,0.043]	-26.8	23.5	0.255	0.985	-0.107	[-0.226,0.013]	-48.0	27.5	0.081	0.405
Frontal Operculum Cortex	ref.	0.013	[-0.092,0.118]	1.6	6.4	0.805	0.985	-0.048	[-0.171,0.075]	-5.7	7.4	0.443	0.717

Lifestyle variable	Sedentary behaviour												
	Low duration	Medium duration						High duration					
Brain structural volumes		Standardised β	95% CI	Unstandardised β	SE	*p*-value	*q*-value	Standardised β	95% CI	Unstandardised β	SE	*p*-value	*q*-value

**Global measures**													
Volume of Grey Matter	ref.	-0.043	[-0.118,0.032]	-520.3	461.4	0.260	0.825	-0.058	[-0.143,0.026]	-704.0	519.4	0.175	0.875
Volume of White Matter	ref.	0.009	[-0.067,0.085]	115.7	522.4	0.825	0.825	0.025	[-0.060,0.111]	342.5	588.1	0.560	0.932
Total Brain Volume	ref.	-0.031	[-0.106,0.043]	-499.2	602.3	0.407	0.825	-0.026	[-0.110,0.058]	-414.4	677.9	0.541	0.932
Volume of White Matter Hyperintensities	ref.	0.034	[-0.044,0.113]	139.1	161.9	0.390	0.825	0.028	[-0.061,0.116]	112.0	182.4	0.539	0.932
**Hippocampal volumes**													
Volume of Left Hippocampus	ref.	0.045	[-0.032,0.122]	9.8	8.5	0.252	0.825	-0.006	[-0.093,0.080]	-1.3	9.6	0.889	0.953
Volume of Right Hippocampus	ref.	0.015	[-0.060,0.090]	3.2	8.3	0.698	0.825	0.000	[-0.085,0.085]	0.0	9.3	0.997	0.997
Hippocampal Asymmetry	ref.	-0.015	[-0.090,0.060]	-4.0	10.0	0.691	0.825	0.017	[-0.067,0.102]	4.5	11.3	0.690	0.932
**Regional GM volumes in Frontal Lobe**													
Frontal Pole	ref.	0.018	[-0.056,0.091]	29.3	62.6	0.640	0.825	0.045	[-0.038,0.128]	74.9	70.4	0.288	0.932
Superior Frontal Gyrus	ref.	0.009	[-0.068,0.086]	8.1	36.6	0.824	0.825	0.014	[-0.072,0.101]	13.4	41.2	0.746	0.932
Middle Frontal Gyrus	ref.	0.030	[-0.046,0.106]	26.0	33.7	0.441	0.825	0.061	[-0.025,0.147]	52.7	38.0	0.166	0.875
Inferior Frontal Gyrus, pars triangularis	ref.	0.010	[-0.067,0.086]	2.2	8.8	0.806	0.825	0.064	[-0.022,0.151]	14.4	9.9	0.145	0.875
Inferior Frontal Gyrus, pars opercularis	ref.	0.010	[-0.067,0.086]	2.1	8.5	0.807	0.825	0.019	[-0.067,0.106]	4.2	9.6	0.661	0.932
Frontal Medial Cortex	ref.	-0.016	[-0.091,0.060]	-3.1	7.5	0.682	0.825	-0.008	[-0.093,0.077]	-1.6	8.5	0.850	0.953
Frontal Orbital Cortex	ref.	0.022	[-0.053,0.096]	9.7	17.1	0.572	0.825	-0.028	[-0.112,0.056]	-12.7	19.3	0.511	0.932
Frontal Operculum Cortex	ref.	-0.010	[-0.087,0.066]	-1.2	4.6	0.796	0.825	0.041	[-0.045,0.127]	4.9	5.2	0.349	0.932

**Lifestyle variable**							**Physical activity**						
	**High METs**			**Medium METs**						**Low METs**			
**Brain structural volumes**		Standardised β	95% CI	Unstandardised β (in mm^3)	SE	p-value	q-value	Standardised β	95% CI	Standardised β	SE	p-value	q-value
**Global measures**													
Volume of Grey Matter	ref.	-0.051	[-0.130,0.028]	-616.2	485.8	0.205	0.384	-0.068	[-0.146,0.011]	-816.8	484.8	0.092	0.69
Volume of White Matter	ref.	0.070	[-0.010,0.150]	940.5	549.9	0.087	0.384	0.054	[-0.026,0.134]	729.2	548.9	0.184	0.757
Total Brain Volume	ref.	0.017	[-0.062,0.095]	264.2	634.2	0.677	0.846	0.000	[-0.079,0.078]	-7.1	633.0	0.991	0.991

Volume of White Matter Hyperintensities	ref.	0.081	[-0.001,0.164]	328.3	170.3	0.054	0.384	0.072	[-0.010,0.155]	292.4	169.9	0.085	0.69
**Hippocampal volumes**													
Volume of Left Hippocampus	ref.	-0.058	[-0.138,0.023]	-12.5	9.0	0.163	0.384	-0.014	[-0.095,0.066]	-3.1	8.9	0.730	0.912
Volume of Right Hippocampus	ref.	-0.004	[-0.083,0.075]	-0.9	8.7	0.916	0.916	0.033	[-0.046,0.112]	7.0	8.7	0.419	0.813
Hippocampal Asymmetry	ref.	0.054	[-0.025,0.133]	14.2	10.5	0.179	0.384	0.045	[-0.034,0.124]	11.8	10.5	0.263	0.757
**Regional GM volumes in Frontal Lobe**													
Frontal Pole	ref.	-0.035	[-0.113,0.042]	-59.1	65.9	0.370	0.555	0.007	[-0.070,0.084]	11.5	65.8	0.861	0.922
Superior Frontal Gyrus	ref.	-0.039	[-0.120,0.042]	-36.4	38.5	0.345	0.555	0.008	[-0.072,0.089]	7.9	38.4	0.838	0.922
Middle Frontal Gyrus	ref.	-0.053	[-0.134,0.027]	-46.1	35.5	0.195	0.384	-0.025	[-0.105,0.055]	-21.7	35.5	0.542	0.813
Inferior Frontal Gyrus, pars triangularis	ref.	-0.024	[-0.105,0.057]	-5.4	9.3	0.557	0.76	-0.030	[-0.110,0.051]	-6.7	9.3	0.470	0.813
Inferior Frontal Gyrus, pars opercularis	ref.	-0.064	[-0.145,0.016]	-14.0	9.0	0.118	0.384	-0.042	[-0.123,0.038]	-9.2	9.0	0.303	0.757
Frontal Medial Cortex	ref.	-0.013	[-0.093,0.066]	-2.6	7.9	0.746	0.861	0.017	[-0.062,0.097]	3.4	7.9	0.666	0.908
Frontal Orbital Cortex	ref.	-0.009	[-0.087,0.070]	-4.0	18.0	0.826	0.885	-0.025	[-0.104,0.053]	-11.4	18.0	0.525	0.813
Frontal Operculum Cortex	ref.	-0.055	[-0.136,0.025]	-6.5	4.9	0.179	0.384	-0.047	[-0.127,0.034]	-5.6	4.9	0.254	0.757

Note: Bold type indicates p <0.05; † denotes indicates q < 0.05 (Benjamini–Hochberg adjusted). Unstandardised βs ((in mm^3) and standardised βs were estimated by linear regression in each lifestyle model, with the reference of each optimal lifestyle, such as, no smoking, low level alcohol drinking, healthy diet, low duration, high METs. Five models were adjusted for corresponding baseline brain structural volume and their z-scores, respectively, *APOE* ε4 genotype, age, sex, genotypic array, MRI sites, genetic principal components, deprivation index, time intervals, intracranial volume among 3265 participants, except for the white matter hyperintensity models (N=3264). CI, confidence interval; SE, standard error; GM, grey matter.

Table S9. Longitudinal associations of single lifestyle variable simultaneously modelled on brain structural changes

**Lifestyle variable**				**Smoking**									
	**Never smoker**			**Former smoker**						**Current smoker**			
**Brain structural volumes**		Standardised *β*	**95% CI**	Unstandardised *β*	SE	*p*-value	*q*-value	Standardised *β*	**95% CI**	Unstandardised *β*	SE	*p*-value	*q*-value
**Global measures**													
Volume of Grey Matter	ref.	0.026	[-0.046,0.098]	313.5	444.6	0.481	0.994	0.009	[-0.176,0.194]	107.2	1140.7	0.925	0.941
Volume of White Matter	ref.	0.015	[-0.058,0.088]	197.6	503.2	0.695	0.994	-0.040	[-0.227,0.148]	-534.0	1291.2	0.679	0.941
Total Brain Volume	ref.	0.038	[-0.034,0.109]	602.8	579.9	0.299	0.994	-0.014	[-0.197,0.170]	-214.9	1488.0	0.885	0.941
Volume of White Matter Hyperintensities	ref.	0.057	[-0.019,0.132]	229.7	155.7	0.140	0.994	**0.200**	**[0.006,0.395]**	**809.9**	**399.7**	**0.043**	0.645
**Hippocampal volumes**													
Volume of Left Hippocampus	ref.	-0.055	[-0.129,0.019]	-11.9	8.2	0.146	0.994	-0.041	[-0.230,0.148]	-8.9	21.0	0.671	0.941
Volume of Right Hippocampus	ref.	-0.045	[-0.117,0.028]	-9.7	8.0	0.225	0.994	0.044	[-0.142,0.230]	9.5	20.4	0.642	0.941
Hippocampal Asymmetry	ref.	0.004	[-0.068,0.076]	1.1	9.6	0.907	0.994	0.088	[-0.097,0.274]	23.1	24.7	0.350	0.941
**Regional GM volumes in Frontal Lobe**													
Frontal Pole	ref.	-0.011	[-0.081,0.060]	-17.5	60.3	0.771	0.994	-0.085	[-0.267,0.097]	-141.6	154.9	0.361	0.941
Superior Frontal Gyrus	ref.	-0.019	[-0.093,0.055]	-17.7	35.3	0.615	0.994	0.077	[-0.113,0.267]	71.5	90.4	0.429	0.941
Middle Frontal Gyrus	ref.	0.035	[-0.038,0.109]	30.7	32.5	0.346	0.994	-0.057	[-0.246,0.132]	-49.6	83.5	0.553	0.941
Inferior Frontal Gyrus, pars triangularis	ref.	0.000	[-0.074,0.074]	0.1	8.5	0.994	0.994	0.055	[-0.134,0.245]	12.5	21.7	0.567	0.941
Inferior Frontal Gyrus, pars opercularis	ref.	-0.031	[-0.105,0.043]	-6.8	8.2	0.410	0.994	-0.035	[-0.224,0.154]	-7.7	21.1	0.716	0.941
Frontal Medial Cortex	ref.	-0.006	[-0.078,0.067]	-1.1	7.3	0.879	0.994	-0.007	[-0.194,0.179]	-1.4	18.6	0.941	0.941
Frontal Orbital Cortex	ref.	0.002	[-0.070,0.074]	1.0	16.5	0.953	0.994	-0.019	[-0.203,0.166]	-8.5	42.3	0.840	0.941
Frontal Operculum Cortex	ref.	0.021	[-0.052,0.095]	2.5	4.5	0.568	0.994	-0.010	[-0.199,0.178]	-1.2	11.4	0.914	0.941

**Lifestyle variable**				**Alcohol drinking**									
	**Low level**			**Moderate level**						**High level**			
**Brain structural volumes**		Standardised *β*	95% CI	Unstandardised *β*	SE	*p*-value	*q*-value	Standardised *β*	95% CI	Unstandardised *β*	SE	*p*-value	*q*-value
**Global measures**													

Brain structural volumes		Standardised β	95% CI	Unstandardised β	SE	p-value	q-value	Standardised β	95% CI	Unstandardised β	SE	p-value	q-value
Volume of Grey Matter	ref.	-0.055	[-0.136,0.026]	-665.0	501.9	0.185	0.706	0.022	[-0.056,0.100]	266.2	482.4	0.581	0.932
Volume of White Matter	ref.	-0.043	[-0.126,0.039]	-582.8	568.8	0.306	0.706	-0.009	[-0.088,0.071]	-119.0	546.2	0.828	0.932
Total Brain Volume	ref.	-0.067	[-0.148,0.014]	-1069.2	655.6	0.103	0.706	0.021	[-0.057,0.098]	329.2	630.1	0.601	0.932
Volume of White Matter Hyperintensities	ref.	0.012	[-0.074,0.097]	47.7	176.1	0.787	0.908	0.010	[-0.072,0.093]	41.9	169.6	0.805	0.932
**Hippocampal volumes**													
Volume of Left Hippocampus	ref.	-0.028	[-0.111,0.056]	-6.0	9.3	0.518	0.706	-0.051	[-0.131,0.029]	-11.0	8.9	0.215	0.932
Volume of Right Hippocampus	ref.	-0.054	[-0.136,0.028]	-11.7	9.0	0.193	0.706	0.003	[-0.075,0.082]	0.7	8.6	0.936	0.936
Hippocampal Asymmetry	ref.	-0.022	[-0.104,0.059]	-5.811	10.9	0.594	0.742	0.050	[-0.028,0.128]	13.1	10.5	0.212	0.932
**Regional GM in Frontal Lobe**													
Frontal Pole	ref.	-0.037	[-0.117,0.043]	-61.3	68.1	0.368	0.706	-0.007	[-0.084,0.070]	-12.2	65.4	0.852	0.932
Superior Frontal Gyrus	ref.	-0.033	[-0.117,0.051]	-30.8	39.8	0.440	0.706	0.011	[-0.069,0.092]	10.6	38.2	0.781	0.932
Middle Frontal Gyrus	ref.	0.030	[-0.053,0.113]	25.9	36.7	0.481	0.706	0.038	[-0.042,0.118]	32.8	35.3	0.353	0.932
Inferior Frontal Gyrus, pars triangularis	ref.	-0.029	[-0.113,0.054]	-6.6	9.6	0.493	0.706	-0.007	[-0.087,0.074]	-1.5	9.2	0.870	0.932
Inferior Frontal Gyrus, pars opercularis	ref.	0.002	[-0.081,0.085]	0.5	9.3	0.960	0.963	0.026	[-0.054,0.107]	5.8	8.9	0.518	0.932
Frontal Medial Cortex	ref.	-0.002	[-0.084,0.080]	-0.4	8.2	0.963	0.963	-0.010	[-0.089,0.069]	-1.9	7.9	0.805	0.932
Frontal Orbital Cortex	ref.	-0.048	[-0.129,0.033]	-21.5	18.6	0.249	0.706	-0.018	[-0.096,0.060]	-8.1	17.9	0.652	0.932
Frontal Operculum Cortex	ref.	-0.048	[-0.131,0.035]	-5.7	5.0	0.259	0.706	-0.008	[-0.088,0.071]	-1.0	4.8	0.836	0.932

**Lifestyle variables**						**Diet**							
	**Healthy diet**			**Moderate diet**						**Unhealthy diet**			
**Brain structural volumes**		Standardised *β*	95% CI	Unstandardised *β*	SE	*p*-value	*q*-value	Standardised *β*	95% CI	Unstandardised *β*	SE	*p*-value	*q*-value
**Global measures**													
Volume of Grey Matter	ref.	0.012	[-0.091,0.115]	143.3	636.8	0.822	0.967	-0.039	[-0.160,0.083]	-467.7	750.0	0.533	0.681
Volume of White Matter	ref.	-0.002	[-0.107,0.103]	-29.8	721.4	0.967	0.967	0.010	[-0.113,0.134]	139.5	849.5	0.870	0.870
Total Brain Volume	ref.	0.005	[-0.098,0.107]	78.1	831.3	0.925	0.967	-0.022	[-0.143,0.098]	-356.7	979.1	0.716	0.767
Volume of White Matter Hyperintensities	ref.	-0.040	[-0.148,0.068]	-162.2	223.1	0.467	0.967	-0.062	[-0.189,0.066]	-249.5	263.0	0.343	0.643
**Hippocampal volumes**													
Volume of Left Hippocampus	ref.	-0.044	[-0.150,0.062]	-9.6	11.7	0.414	0.967	-0.113	[-0.238,0.012]	-24.6	13.8	0.075	0.450

Volume of Right Hippocampus	ref.	-0.059	[-0.163,0.045]	-12.8	11.4	0.263	0.967	-0.038	[-0.160,0.085]	-8.1	13.4	0.545	0.681
Hippocampal Asymmetry (Right-Left)	ref.	0.003	[-0.101,0.106]	0.7	13.8	0.962	0.967	0.064	[-0.058,0.186]	16.8	16.3	0.303	0.643
**Regional GM volumes in Frontal Lobe**													
Frontal Pole	ref.	0.003	[-0.099,0.104]	4.4	86.4	0.959	0.967	-0.059	[-0.179,0.060]	-98.9	101.8	0.331	0.643
Superior Frontal Gyrus	ref.	-0.031	[-0.137,0.075]	-28.9	50.5	0.567	0.967	-0.104	[-0.229,0.021]	-97.2	59.5	0.102	0.450
Middle Frontal Gyrus	ref.	0.019	[-0.086,0.125]	16.8	46.6	0.718	0.967	-0.039	[-0.163,0.085]	-33.5	54.9	0.541	0.681
Inferior Frontal Gyrus, pars triangularis	ref.	0.011	[-0.095,0.117]	2.5	12.1	0.835	0.967	-0.092	[-0.216,0.033]	-20.6	14.3	0.150	0.450
Inferior Frontal Gyrus, pars opercularis	ref.	0.041	[-0.064,0.147]	9.0	11.8	0.442	0.967	-0.028	[-0.152,0.097]	-6.0	13.9	0.664	0.766
Frontal Medial Cortex	ref.	-0.054	[-0.158,0.051]	-10.5	10.4	0.313	0.967	**-0.124**	**[-0.247, -0.001]**	**-24.3**	**12.3**	**0.048**	0.450
Frontal Orbital Cortex	ref.	-0.054	[-0.157,0.049]	-24.4	23.6	0.301	0.967	-0.096	[-0.217,0.026]	-43.0	27.8	0.123	0.450
Frontal Operculum Cortex	ref.	0.011	[-0.095,0.116]	1.3	6.4	0.838	0.967	-0.051	[-0.175,0.073]	-6.1	7.5	0.419	0.681

**Lifestyle variables**	**Sedentary behaviour**												
	**Low duration**	**Medium duration**						**High duration**					
**Brain structural volumes**		Standardised β	**95% CI**	Unstandardised β	SE	*p*-value	*q*-value	Standardised β	**95% CI**	Unstandardised β	SE	*p*-value	*q*-value
**Global measures**													
Volume of Grey Matter	ref.	-0.040	[-0.115,0.035]	-483.1	462.4	0.296	0.869	-0.053	[-0.138,0.033]	-635.6	526.4	0.227	0.825
Volume of White Matter	ref.	0.007	[-0.069,0.083]	91.2	523.8	0.862	0.869	0.023	[-0.064,0.109]	307.1	596.4	0.607	0.938
Total Brain Volume	ref.	-0.031	[-0.106,0.043]	-500.2	603.8	0.408	0.869	-0.026	[-0.111,0.058]	-419.8	687.4	0.541	0.938
Volume of White Matter Hyperintensities	ref.	0.031	[-0.048,0.109]	124.1	162.2	0.444	0.869	0.020	[-0.069,0.110]	82.1	184.6	0.656	0.938
**Hippocampal volumes**													
Volume of Left Hippocampus	ref.	0.050	[-0.027,0.127]	10.9	8.5	0.200	0.869	0.009	[-0.079,0.096]	1.9	9.7	0.843	0.987
Volume of Right Hippocampus	ref.	0.015	[-0.060,0.091]	3.3	8.3	0.693	0.869	0.001	[-0.085,0.087]	0.2	9.4	0.987	0.987
Hippocampal Asymmetry	ref.	-0.019	[-0.095,0.056]	-5.073	10.0	0.613	0.869	0.005	[-0.081,0.091]	1.3	11.4	0.907	0.987
**Regional GM volumes in Frontal Lobe**													
Frontal Pole	ref.	0.020	[-0.054,0.093]	32.9	62.7	0.600	0.869	0.050	[-0.034,0.134]	84.0	71.4	0.240	0.825
Superior Frontal Gyrus	ref.	0.011	[-0.066,0.088]	10.2	36.7	0.782	0.869	0.019	[-0.069,0.107]	17.7	41.8	0.672	0.938
Middle Frontal Gyrus	ref.	0.030	[-0.047,0.107]	26.0	33.8	0.442	0.869	0.060	[-0.027,0.147]	52.1	38.5	0.176	0.825
Inferior Frontal Gyrus, pars triangularis	ref.	0.014	[-0.063,0.091]	3.2	8.8	0.721	0.869	0.075	[-0.012,0.163]	16.9	10.0	0.093	0.825

Inferior Frontal Gyrus, pars opercularis	ref.	0.013	[-0.064,0.090]	2.8	8.5	0.742	0.869	0.025	[-0.062,0.113]	5.5	9.7	0.573	0.938
Frontal Medial Cortex	ref.	-0.014	[-0.090,0.061]	-2.8	7.6	0.713	0.869	-0.001	[-0.087,0.085]	-0.2	8.6	0.978	0.987
Frontal Orbital Cortex	ref.	0.025	[-0.050,0.100]	11.2	17.2	0.516	0.869	-0.017	[-0.103,0.068]	-7.8	19.5	0.688	0.938
Frontal Operculum Cortex	ref.	-0.006	[-0.083,0.070]	-0.8	4.6	0.869	0.869	0.049	[-0.039,0.136]	5.8	5.3	0.275	0.825

**Lifestyle variables**						**Physical activity**							
	**High METs**			**Medium METs**						**Low METs**			
**Brain structural phenotypes**		Standardised *β*	**95% CI**	Unstandardised *β*	SE	*p*-value	*q*-value	Standardised *β*	**95% CI**	Unstandardised *β*	SE	*p*-value	*q*-value
**Global measures**													
Volume of Grey Matter	ref.	-0.052	[-0.131,0.027]	-628.2	486.3	0.197	0.369	-0.060	[-0.140,0.019]	-729.6	488.4	0.135	0.819
Volume of White Matter	ref.	0.068	[-0.012,0.148]	923.0	550.9	0.094	0.369	0.051	[-0.029,0.132]	692.0	553.4	0.211	0.819
Total Brain Volume	ref.	0.015	[-0.063,0.094]	245.1	634.9	0.699	0.823	0.004	[-0.075,0.082]	56.0	637.7	0.930	0.93
Volume of White Matter Hyperintensities	ref.	0.082	[-0.001,0.165]	330.6	170.5	0.052	0.369	0.074	[-0.009,0.157]	300.5	171.1	0.079	0.819
**Hippocampal volumes**													
Volume of Left Hippocampus	ref.	-0.062	[-0.143,0.019]	-13.4	9.0	0.135	0.369	-0.010	[-0.091,0.071]	-2.2	9.0	0.810	0.927
Volume of Right Hippocampus	ref.	-0.006	[-0.085,0.074]	-1.2	8.7	0.887	0.887	0.031	[-0.049,0.111]	6.7	8.7	0.446	0.819
Hippocampal Asymmetry	ref.	0.056	[-0.023,0.135]	14.7	10.6	0.163	0.369	0.039	[-0.040,0.119]	10.3	10.6	0.330	0.819
**Regional GM volumes in Frontal Lobe**													
Frontal Pole	ref.	-0.038	[-0.116,0.039]	-63.7	66.0	0.335	0.503	0.007	[-0.071,0.085]	11.3	66.3	0.865	0.927
Superior Frontal Gyrus	ref.	-0.041	[-0.122,0.040]	-38.0	38.6	0.324	0.503	0.012	[-0.070,0.093]	11.1	38.7	0.774	0.927
Middle Frontal Gyrus	ref.	-0.053	[-0.134,0.027]	-46.1	35.6	0.195	0.369	-0.025	[-0.106,0.056]	-21.6	35.7	0.545	0.819
Inferior Frontal Gyrus, pars triangularis	ref.	-0.026	[-0.107,0.055]	-5.9	9.3	0.523	0.713	-0.029	[-0.111,0.052]	-6.6	9.3	0.477	0.819
Inferior Frontal Gyrus, pars opercularis	ref.	-0.065	[-0.146,0.016]	-14.2	9.0	0.114	0.369	-0.042	[-0.123,0.040]	-9.1	9.0	0.315	0.819
Frontal Medial Cortex	ref.	-0.014	[-0.094,0.065]	-2.8	8.0	0.722	0.823	0.025	[-0.055,0.105]	4.8	8.0	0.546	0.819
Frontal Orbital Cortex	ref.	-0.012	[-0.091,0.067]	-5.3	18.1	0.768	0.823	-0.020	[-0.099,0.059]	-8.9	18.1	0.625	0.852
Frontal Operculum Cortex	ref.	-0.057	[-0.138,0.023]	-6.8	4.9	0.165	0.369	-0.047	[-0.127,0.034]	-5.5	4.9	0.259	0.819

Note: Bold type indicates p <0.05; † denotes indicates q < 0.05 (Benjamini–Hochberg adjusted). Unstandardised βs ((in mm^3) and standardised βs were estimated by linear regression in the simultaneous lifestyle model, with the reference of each optimal lifestyle, such as, no smoking, low level alcohol drinking, healthy diet, low duration, high METs. Model was adjusted for corresponding baseline brain structural volume and their z-scores, respectively, all lifestyles, *APOE* ε4 genotype, age, sex, genotypic array, MRI sites, genetic principal components, deprivation index, time intervals, intracranial volume among 3265 participants, except for the white matter hyperintensity models (N=3264). CI, confidence interval; SE, standard error; GM, grey matter.

TableS10. Two-way interactions of lifestyle categories × *APOE* ε4 genotype on longitudinal brain structural changes

Brain structural volumes	*APOE* ε4 presence × Unfavourable lifestyle						*APOE* ε4 presence × Moderate lifestyle					
	Standardised *β*	95% CI	Unstandardised *β*	SE	*p*-value	*q*-value	Standardised *β*	95% CI	Unstandardised *β*	SE	*p*-value	*q*-value
**Global measures**												
Volume of Grey Matter	-0.054	[-0.311,0.203]	-654.0	1585.3	0.680	0.978	-0.059	[-0.219,0.100]	-718.8	983.4	0.465	0.896
Volume of White Matter	0.074	[-0.187,0.335]	995.8	1794.9	0.579	0.978	0.045	[-0.117,0.207]	609.6	1113.1	0.584	0.896
Total Brain Volume	0.006	[-0.250,0.261]	88.6	2069.7	0.966	0.978	-0.001	[-0.159,0.157]	-16.0	1283.2	0.990	0.99
Volume of White Matter Hyperintensities	0.088	[-0.181,0.358]	357.1	555.7	0.521	0.978	-0.066	[-0.233,0.102]	-265.8	344.7	0.441	0.896
**Hippocampal volumes**												
Volume of Left Hippocampus	0.032	[-0.231,0.295]	7.0	29.2	0.812	0.978	-0.047	[-0.210,0.116]	-10.2	18.1	0.573	0.896
Volume of Right Hippocampus	0.014	[-0.245,0.272]	2.9	28.4	0.917	0.978	0.022	[-0.138,0.183]	4.8	17.6	0.786	0.896
Hippocampal Asymmetry (Right-Left)	-0.004	[-0.261,0.254]	-1.0	34.4	0.978	0.978	0.059	[-0.101,0.219]	15.5	21.3	0.467	0.896
**Regional GM volumes in Frontal Lobe**												
Frontal Pole	0.185	[-0.068,0.437]	308.2	215.0	0.152	0.978	0.079	[-0.077,0.236]	132.4	133.3	0.321	0.896
Superior Frontal Gyrus	0.096	[-0.169,0.360]	89.3	125.8	0.478	0.978	0.036	[-0.128,0.200]	33.6	78.0	0.666	0.896
Middle Frontal Gyrus	0.056	[-0.207,0.318]	48.3	116.0	0.677	0.978	0.081	[-0.082,0.243]	69.9	71.9	0.331	0.896
Inferior Frontal Gyrus, pars triangularis	0.039	[-0.225,0.303]	8.8	30.3	0.772	0.978	-0.03	[-0.194,0.134]	-6.7	18.8	0.722	0.896
Inferior Frontal Gyrus, pars opercularis	0.081	[-0.182,0.345]	17.7	29.3	0.545	0.978	0.055	[-0.108,0.218]	12.0	18.2	0.507	0.896
Frontal Medial Cortex	0.088	[-0.172,0.347]	17.1	25.9	0.508	0.978	0.036	[-0.124,0.197]	7.1	16.1	0.657	0.896
Frontal Orbital Cortex	0.089	[-0.167,0.346]	40.2	58.8	0.495	0.978	0.017	[-0.142,0.176]	7.6	36.5	0.836	0.896
Frontal Operculum Cortex	0.111	[-0.151,0.374]	13.2	15.9	0.406	0.978	-0.033	[-0.196,0.130]	-3.9	9.9	0.691	0.896

Note: Bold type indicates p <0.05; † denotes indicates q < 0.05 (Benjamini–Hochberg adjusted). Unstandardised *β*s ((in mm^3) and standardised *β*s were estimated by linear regression models including interaction terms between *APOE* ε4 genotype and lifestyle categories. Models were adjusted for corresponding baseline brain structural volume

and their z-scores respectively, age, sex, genotypic array, MRI sites, genetic principal components, deprivation index, time intervals, intracranial volume among 3265 participants, except for the white matter hyperintensity models (N=3264). CI, confidence interval; SE, standard error.

Table S11. Two-way interactions of each lifestyle variable × *APOE* ε4 genotype on longitudinal brain structural changes

	APOE ε4 presence × Current smoker						*APOE* ε4 presence × Former smoker					
**Brain structural volumes**	Standardised *β*	95% CI	Unstandardised *β*	SE	*p*-value	*q*-value	Standardised *β*	95% CI	Unstandardised *β*	SE	*p*-value	*q*-value
**Global measures**												
Volume of Grey Matter	-0.011	[-0.422,0.400]	-134.7	2534.1	0.958	0.994	-0.042	[-0.204,0.121]	-501.9	1000.1	0.616	0.934
Volume of White Matter	0.002	[-0.415,0.419]	22.4	2868.2	0.994	0.994	-0.053	[-0.218,0.111]	-720.5	1131.8	0.524	0.934
Total Brain Volume	-0.002	[-0.410,0.405]	-37.1	3306.1	0.991	0.994	-0.07	[-0.231,0.090]	-1119.6	1304.7	0.391	0.934
Volume of White Matter Hyperintensities	0.004	[-0.427,0.434]	14.4	887.5	0.987	0.994	-0.007	[-0.177,0.163]	-28.0	350.3	0.936	0.934
**Hippocampal volumes**												
Volume of Left Hippocampus	-0.226	[-0.647,0.195]	-49.1	46.7	0.293	0.994	0.092	[-0.075,0.258]	19.9	18.4	0.280	0.934
Volume of Right Hippocampus	-0.16	[-0.572,0.253]	-34.3	45.3	0.449	0.994	0.045	[-0.118,0.208]	9.7	17.9	0.589	0.934
Hippocampal Asymmetry	0.085	[-0.327,0.497]	22.3	55.0	0.685	0.994	-0.039	[-0.201,0.124]	-10.1	21.7	0.641	0.934
**Regional GM volumes in Frontal Lobe**												
Frontal Pole	0.068	[-0.336,0.472]	113.4	343.6	0.741	0.994	-0.016	[-0.176,0.143]	-27.2	135.6	0.841	0.934
Superior Frontal Gyrus	0.211	[-0.212,0.633]	196.4	200.9	0.328	0.994	0.038	[-0.129,0.205]	35.3	79.3	0.656	0.934
Middle Frontal Gyrus	-0.066	[-0.485,0.353]	-57.2	185.3	0.757	0.994	0.136	[-0.030,0.301]	117.7	73.1	0.108	0.934
Inferior Frontal Gyrus, pars triangularis	0.134	[-0.288,0.555]	30.0	48.3	0.535	0.994	0.041	[-0.125,0.208]	9.3	19.1	0.625	0.934
Inferior Frontal Gyrus, pars opercularis	0.154	[-0.267,0.574]	33.5	46.8	0.474	0.994	0.113	[-0.053,0.279]	24.6	18.5	0.183	0.934
Frontal Medial Cortex	0.064	[-0.351,0.478]	12.5	41.4	0.763	0.994	-0.032	[-0.195,0.132]	-6.2	16.3	0.705	0.934
Frontal Orbital Cortex	0.172	[-0.238,0.582]	77.3	94.0	0.411	0.994	0.006	[-0.156,0.168]	2.7	37.1	0.942	0.942
Frontal Operculum Cortex	0.154	[-0.265,0.574]	18.4	25.4	0.47	0.994	0.024	[-0.141,0.190]	2.9	10.0	0.776	0.934

	APOE ε4 presence × High risk						*APOE* ε4 presence × Moderate risk					
**Brain structural volumes**	Standardised *β*	95% CI	Unstandardised *β*	SE	*p*-value	*q*-value	Standardised *β*	95% CI	Unstandardised *β*	SE	*p*-value	*q*-value
**Global measures**												
Volume of Grey Matter	-0.035	[-0.217,0.147]	-426.0	1120.2	0.704	0.943	-0.148	[-0.326,0.029]	-1793.6	1096.1	0.102	0.628
Volume of White Matter	0.117	[-0.067,0.302]	1581.9	1268.5	0.212	0.943	0.100	[-0.080,0.281]	1352.3	1241.7	0.276	0.628
Total Brain Volume	0.070	[-0.110,0.250]	1110.4	1461.7	0.448	0.943	-0.040	[-0.217,0.136]	-640.3	1429.0	0.654	0.811
Volume of White Matter Hyperintensities	0.087	[-0.104,0.277]	349.5	393.0	0.374	0.943	-0.052	[-0.238,0.135]	-209.9	384.2	0.585	0.811
**Hippocampal volumes**												
Volume of Left Hippocampus	0.007	[-0.180,0.193]	1.5	20.7	0.943	0.943	-0.006	[-0.188,0.176]	-1.3	20.2	0.950	0.971
Volume of Right Hippocampus	0.016	[-0.166,0.199]	3.5	20.1	0.861	0.943	0.113	[-0.066,0.291]	24.3	19.6	0.216	0.628

Hippocampal Asymmetry	-0.007	[-0.190,0.175]	-1.9	24.3	0.936	0.943	0.093	[-0.085,0.271]	24.4	23.8	0.305	0.628
**Regional GM volumes in Frontal Lobe**												
Frontal Pole	-0.031	[-0.209,0.148]	-51.5	152.0	0.735	0.943	0.106	[-0.069,0.281]	176.9	148.6	0.234	0.628
Superior Frontal Gyrus	-0.072	[-0.259,0.115]	-67.4	88.8	0.448	0.943	0.064	[-0.119,0.246]	59.3	86.9	0.495	0.811
Middle Frontal Gyrus	-0.009	[-0.195,0.176]	-8.2	82.0	0.921	0.943	0.035	[-0.146,0.217]	30.6	80.1	0.703	0.811
Inferior Frontal Gyrus, pars triangularis	-0.114	[-0.301,0.072]	-25.6	21.4	0.230	0.943	0.090	[-0.093,0.272]	20.2	20.9	0.335	0.628
Inferior Frontal Gyrus, pars opercularis	0.020	[-0.166,0.206]	4.3	20.7	0.834	0.943	0.093	[-0.089,0.275]	20.2	20.2	0.318	0.628
Frontal Medial Cortex	-0.038	[-0.222,0.145]	-7.5	18.3	0.683	0.943	-0.003	[-0.183,0.176]	-0.7	17.9	0.971	0.971
Frontal Orbital Cortex	-0.115	[-0.296,0.066]	-51.6	41.5	0.214	0.943	0.107	[-0.070,0.284]	48.1	40.6	0.237	0.628
Frontal Operculum Cortex	-0.063	[-0.249,0.122]	-7.5	11.2	0.505	0.943	-0.042	[-0.224,0.139]	-5.0	11.0	0.647	0.811

	APOE ε4 presence × Unhealthy diet						*APOE* ε4 presence × Moderate diet					
**Brain structural volumes**	Standardised *β*	95% CI	Unstandardised *β*	SE	*p*-value	*q*-value	Standardised *β*	95% CI	Unstandardised *β*	SE	*p*-value	*q*-value
**Global measures**												
Volume of Grey Matter	0.075	[-0.190,0.341]	910.1	1636.8	0.578	0.995	0.005	[-0.225,0.235]	64.1	1417.4	0.964	0.964
Volume of White Matter	-0.032	[-0.301,0.237]	-434.0	1852.5	0.815	0.995	-0.193	[-0.426,0.040]	-2601.0	1603.7	0.105	0.935
Total Brain Volume	-0.001	[-0.264,0.263]	-13.4	2135.7	0.995	0.995	-0.175	[-0.403,0.053]	-2783.6	1848.8	0.132	0.935
Volume of White Matter Hyperintensities	0.024	[-0.255,0.303]	95.9	574.5	0.867	0.995	-0.057	[-0.298,0.184]	-229.9	497.1	0.644	0.935
**Hippocampal volumes**												
Volume of Left Hippocampus	-0.133	[-0.405,0.139]	-29.0	30.2	0.337	0.995	-0.043	[-0.279,0.192]	-9.4	26.1	0.719	0.935
Volume of Right Hippocampus	-0.147	[-0.414,0.120]	-31.6	29.3	0.280	0.995	0.020	[-0.211,0.251]	4.4	25.4	0.862	0.935
Hippocampal Asymmetry	0.021	[-0.245,0.287]	5.6	35.5	0.876	0.995	0.053	[-0.177,0.284]	13.9	30.7	0.650	0.935
**Regional GM volumes in Frontal Lobe**												
Frontal Pole	0.141	[-0.120,0.402]	235.5	222.0	0.289	0.995	0.074	[-0.152,0.300]	123.3	192.2	0.521	0.935
Superior Frontal Gyrus	-0.017	[-0.289,0.256]	-15.4	129.8	0.905	0.995	0.037	[-0.199,0.273]	34.5	112.4	0.759	0.935
Middle Frontal Gyrus	-0.086	[-0.357,0.184]	-74.9	119.7	0.531	0.995	0.127	[-0.107,0.362]	110.2	103.6	0.287	0.935
Inferior Frontal Gyrus, pars triangularis	-0.011	[-0.283,0.261]	-2.4	31.2	0.938	0.995	0.046	[-0.190,0.281]	10.2	27.0	0.705	0.935
Inferior Frontal Gyrus, pars opercularis	-0.018	[-0.289,0.254]	-3.9	30.2	0.899	0.995	0.107	[-0.128,0.342]	23.3	26.2	0.372	0.935
Frontal Medial Cortex	-0.058	[-0.326,0.210]	-11.4	26.7	0.671	0.995	-0.019	[-0.251,0.213]	-3.7	23.1	0.873	0.935
Frontal Orbital Cortex	-0.104	[-0.369,0.160]	-47.0	60.7	0.439	0.995	-0.045	[-0.274,0.184]	-20.2	52.6	0.700	0.935
Frontal Operculum Cortex	-0.071	[-0.342,0.200]	-8.4	16.4	0.608	0.995	-0.042	[-0.277,0.193]	-5.0	14.2	0.726	0.935

	APOE ε4 presence × High sedentary						*APOE* ε4 presence × Medium sedentary					
**Brain structural volumes**	Standardised *β*	**95% CI**	Unstandardised *β*	SE	P-values	q-value	Standardised *β*	**95% CI**	Unstandardised *β*	SE	P-values	q-value

**Global measures**												
Volume of Grey Matter	-0.120	[-0.311,0.070]	-1456.0	1172.1	0.214	0.718	-0.086	[-0.258,0.086]	-1037.9	1060.8	0.328	0.776
Volume of White Matter	0.031	[-0.162,0.224]	422.7	1327.5	0.750	0.915	0.066	[-0.109,0.240]	887.5	1201.2	0.460	0.776
Total Brain Volume	-0.077	[-0.266,0.111]	-1230.1	1530.3	0.422	0.718	-0.008	[-0.178,0.163]	-122.3	1384.7	0.930	0.981
Volume of White Matter Hyperintensities	-0.092	[-0.291,0.108]	-370.4	411.2	0.368	0.718	-0.060	[-0.241,0.121]	-242.6	372.1	0.515	0.776
**Hippocampal volumes**												
Volume of Left Hippocampus	0.093	[-0.102,0.288]	20.2	21.6	0.349	0.718	-0.055	[-0.232,0.121]	-12.0	19.6	0.539	0.776
Volume of Right Hippocampus	0.110	[-0.081,0.301]	23.7	21.0	0.259	0.718	0.047	[-0.126,0.220]	10.0	19.0	0.598	0.776
Hippocampal Asymmetry	0.009	[-0.181,0.200]	2.5	25.4	0.923	0.923	0.081	[-0.092,0.253]	21.2	23.0	0.358	0.776
**Regional GM volumes in Frontal Lobe**												
Frontal Pole	0.084	[-0.103,0.271]	140.0	159.0	0.379	0.718	0.134	[-0.036,0.303]	222.8	143.9	0.122	0.776
Superior Frontal Gyrus	0.128	[-0.068,0.323]	118.9	93.0	0.201	0.718	0.002	[-0.175,0.179]	2.0	84.2	0.981	0.981
Middle Frontal Gyrus	0.026	[-0.168,0.220]	22.5	85.7	0.793	0.915	0.134	[-0.042,0.309]	116.0	77.6	0.135	0.776
Inferior Frontal Gyrus, pars triangularis	0.130	[-0.065,0.325]	29.2	22.4	0.191	0.718	0.051	[-0.125,0.228]	11.5	20.2	0.569	0.776
Inferior Frontal Gyrus, pars opercularis	-0.015	[-0.210,0.180]	-3.2	21.7	0.882	0.923	0.063	[-0.113,0.239]	13.7	19.6	0.483	0.776
Frontal Medial Cortex	0.077	[-0.115,0.269]	15.1	19.2	0.431	0.718	0.044	[-0.130,0.218]	8.6	17.4	0.621	0.776
Frontal Orbital Cortex	0.031	[-0.159,0.221]	13.8	43.5	0.750	0.915	0.019	[-0.153,0.191]	8.6	39.4	0.828	0.955
Frontal Operculum Cortex	0.050	[-0.144,0.244]	5.9	11.8	0.614	0.915	0.103	[-0.072,0.279]	12.3	10.6	0.248	0.776

	APOE ε4 presence × Low METs						*APOE* ε4 presence × Medium METs					
**Brain structural volumes**	Standardised β	95% CI	Unstandardised β	SE	*p*-value	*q*-value	Standardised β	95% CI	Unstandardised β	SE	*p*-value	*q*-value
**Global measures**												
Volume of Grey Matter	-0.047	[-0.228,0.134]	-570.9	1115.9	0.609	0.992	0.075	[-0.108,0.259]	909.3	1131.1	0.421	0.727
Volume of White Matter	-0.046	[-0.230,0.138]	-621.1	1263.2	0.623	0.992	-0.074	[-0.260,0.112]	-998.4	1280.4	0.436	0.727
Total Brain Volume	-0.072	[-0.251,0.108]	-1138.4	1456.8	0.435	0.992	0.000	[-0.182,0.182]	2.1	1476.8	0.999	0.999
Volume of White Matter Hyperintensities	0.030	[-0.160,0.220]	121.2	391.1	0.757	0.992	0.078	[-0.115,0.270]	313.8	396.7	0.429	0.727
**Hippocampal volumes**												
Volume of Left Hippocampus	-0.003	[-0.189,0.182]	-0.7	20.6	0.973	0.992	-0.142	[-0.330,0.046]	-30.9	20.9	0.139	0.727
Volume of Right Hippocampus	-0.047	[-0.229,0.135]	-10.1	20.0	0.613	0.992	-0.115	[-0.300,0.069]	-24.8	20.3	0.221	0.727
Hippocampal Asymmetry	-0.026	[-0.208,0.155]	-6.8	24.2	0.778	0.992	0.013	[-0.171,0.197]	3.3	24.5	0.893	0.963
**Regional GM volumes in Frontal Lobe**												
Frontal Pole	0.001	[-0.177,0.179]	1.4	151.4	0.992	0.992	-0.094	[-0.275,0.086]	-157.4	153.4	0.305	0.727
Superior Frontal Gyrus	0.084	[-0.102,0.270]	78.1	88.5	0.378	0.992	-0.012	[-0.201,0.176]	-11.4	89.7	0.899	0.963

Middle Frontal Gyrus	-0.024	[-0.209,0.161]	-20.8	81.6	0.799	0.992	-0.099	[-0.286,0.088]	-85.9	82.8	0.300	0.727
Inferior Frontal Gyrus, pars triangularis	-0.083	[-0.269,0.103]	-18.6	21.3	0.382	0.992	-0.123	[-0.312,0.065]	-27.7	21.6	0.199	0.727
Inferior Frontal Gyrus, pars opercularis	0.019	[-0.167,0.204]	4.1	20.6	0.844	0.992	-0.041	[-0.228,0.147]	-8.8	20.9	0.672	0.916
Frontal Medial Cortex	0.028	[-0.154,0.211]	5.5	18.2	0.762	0.992	-0.027	[-0.213,0.158]	-5.4	18.5	0.771	0.963
Frontal Orbital Cortex	0.021	[-0.160,0.202]	9.4	41.4	0.820	0.992	-0.054	[-0.237,0.129]	-24.2	42.0	0.564	0.846
Frontal Operculum Cortex	0.011	[-0.173,0.196]	1.4	11.2	0.903	0.992	-0.113	[-0.300,0.074]	-13.5	11.4	0.236	0.727

Note: Bold type indicates p <0.05; † denotes indicates q < 0.05 (Benjamini–Hochberg adjusted). Unstandardised βs ((in mm^3) and standardised βs were estimated by linear regression in each lifestyle model including interaction terms between each lifestyle variable and *APOE* ε4 genotype. Models were adjusted for corresponding baseline brain structural volume and their z-scores respectively, age, sex, genotypic array, MRI sites, genetic principal components, deprivation index, time intervals, intracranial volume among 3265 participants, except for the white matter hyperintensity models (N=3264). CI, confidence interval; SE, standard error; GM, grey matter.

Table S12. Two-way interactions of sex × *APOE* ε4 genotype on longitudinal brain structural changes among participants aged ≤60 years

	APOE ε4 presence × Female					
**Brain structural phenotypes**	Unstandardised β	**SE**	standardised β	95% CI	p-value	q-value
**Global measures**						
Volume of Grey Matter	60.9	1300.6	0.005	[-0.206,0.216]	0.963	0.991
Volume of White Matter	-12.9	1119.2	-0.001	[-0.164,0.162]	0.991	0.991
Total Brain Volume	-47.5	1646.8	-0.003	[-0.206,0.200]	0.977	0.991
Volume of White Matter Hyperintensities	-11.6	-11.6			0.97	0.991
**Hippocampal volumes**						
Volume of Left Hippocampus	-9.3	23.8	-0.043	[-0.258,0.172]	0.695	0.948
Volume of Right Hippocampus	-23.5	23.7	-0.109	[-0.326,0.107]	0.322	0.604
Hippocampal Asymmetry (Right-Left)	-16.2	29.0	-0.062	[-0.279,0.155]	0.576	0.864
**Regional grey matter volumes in Frontal Lobe**						
Grey Matter in Frontal Pole	-281.7	188.9	-0.169	[-0.391,0.053]	0.136	0.408
Grey Matter in Superior Frontal Gyrus	**-300.8**	**111.3**	**-0.323**	**[-0.557,-0.089]**	**0.007**	0.105
Grey Matter in Middle Frontal Gyrus	-137.7	101.2	-0.159	[-0.388,0.070]	0.174	0.435
Grey Matter in Inferior Frontal Gyrus, pars triangularis	-42.4	25.9	-0.189	[-0.414,0.037]	0.101	0.379
Grey Matter in Inferior Frontal Gyrus, pars opercularis	**-57.6**	**25.2**	**-0.264**	**[-0.491,-0.037]**	**0.023**	0.172
Grey Matter in Frontal Medial Cortex	-16.6	22.7	-0.085	[-0.311,0.142]	0.465	0.775
Grey Matter in Frontal Orbital Cortex	-86.2	50.2	-0.192	[-0.411,0.027]	0.086	0.379
Grey Matter in Frontal Operculum Cortex	-18.1	14.5	-0.152	[-0.392,0.087]	0.213	0.456

Note: Bold type indicates p <0.05; † denotes indicates q < 0.05 (Benjamini–Hochberg adjusted). Unstandardised βs ((in mm^3) and standardised βs were estimated by linear regression in younger participants including interaction terms between sex and *APOE* ε4 genotype. Models were adjusted for corresponding baseline brain structural volume and their z-scores respectively, age, genotypic array, MRI sites, genetic principal components, deprivation index, time intervals, intracranial volume among 3265 participants, except for the white matter hyperintensity models (N=3264). CI, confidence interval; SE, standard error; GM, grey matter.

Table S13. Two-way interactions of sex × *APOE* ε4 genotype on longitudinal brain structural changes among participants aged >60 years old

	APOE ε4 presence × Female					
**Brain structural phenotypes**	Unstandardised *β*	**SE**	Standardised *β*	95% CI	*p*-value	*q*-value
**Global measures**						
Volume of Grey Matter	2020.7	1313.7	0.167	[-0.046,0.380]	0.124	0.266
Volume of White Matter	-696.9	1721.6	-0.052	[-0.302,0.199]	0.686	0.686
Total Brain Volume	945.7	1760.1	0.059	[-0.158,0.277]	0.591	0.678
Volume of White Matter Hyperintensities	523.9	554.0	0.13	[-0.139,0.399]	0.344	0.573
**Hippocampal volumes**						
Volume of Left Hippocampus	18.1	24.3	0.083	[-0.136,0.303]	0.455	0.678
Volume of Right Hippocampus	33.2	23.1	0.154	[-0.056,0.365]	0.151	0.283
Hippocampal Asymmetry (Right-Left)	15.9	28.0	0.061	[-0.149,0.271]	0.571	0.678
**Regional grey matter volumes in Frontal Lobe**						
Grey Matter in Frontal Pole	79.5	166.3	0.048	[-0.148,0.243]	0.633	0.678
Grey Matter in Superior Frontal Gyrus	**221.4**	**96.7**	**0.237**	**[0.034,0.441]**	**0.022**	0.101
Grey Matter in Middle Frontal Gyrus	**188.5**	**91.0**	**0.218**	**[0.012,0.423]**	**0.038**	0.114
Grey Matter in Inferior Frontal Gyrus, pars triangularis	**53.7**	**24.2**	**0.239**	**[0.028,0.450]**	**0.027**	0.101
Grey Matter in Inferior Frontal Gyrus, pars opercularis	**56.3**	**23.2**	**0.258**	**[0.050,0.466]**	**0.015**	0.101
Grey Matter in Frontal Medial Cortex	12.3	20.4	0.063	[-0.141,0.267]	0.546	0.678
Grey Matter in Frontal Orbital Cortex	78.7	47.2	0.175	[-0.031,0.381]	0.095	0.238
Grey Matter in Frontal Operculum Cortex	**32.0**	**11.8**	**0.269**	**[0.074,0.464]**	**0.007**	0.101

Note: Bold type indicates p <0.05; † denotes indicates q < 0.05 (Benjamini–Hochberg adjusted). Unstandardised βs ((in mm^3) and standardised βs were estimated by linear regression in old participants including interaction terms between sex and *APOE* ε4 genotype. Models were adjusted for corresponding baseline brain structural volume and their z-scores respectively, age, genotypic array, MRI sites, genetic principal components, deprivation index, time intervals, intracranial volume among 3265 participants, except for the white matter hyperintensity models (N=3264). CI, confidence interval; SE, standard error; GM, grey matter.
